# Recent Advances in Poly(α-*L*-glutamic acid)-Based Nanomaterials for Drug Delivery

**DOI:** 10.3390/biom12050636

**Published:** 2022-04-25

**Authors:** Yu Zhang, Wenliang Song, Yiming Lu, Yixin Xu, Changping Wang, Deng-Guang Yu, Il Kim

**Affiliations:** 1 School of Pharmacy, Shanghai University of Medicine & Health Sciences, Shanghai 201318, China; zhangy_21@sumhs.edu.cn (Y.Z.); b21070102030@stu.sumhs.edu.cn (Y.L.); xuyx@sumhs.edu.cn (Y.X.); 2 Department of Polymer Science and Engineering, Pusan National University, Busan 46241, Korea; wenliang@usst.edu.cn; 3 School of Materials Science & Engineering, University of Shanghai for Science and Technology, Shanghai 200093, China; 1935040425@st.usst.edu.cn

**Keywords:** cancer therapy, drug delivery system, nanomaterials, prodrugs, poly(*α*-*L*-glutamic acid)

## Abstract

Poly(*α*-*L*-glutamic acid) (PGA) is a class of synthetic polypeptides composed of the monomeric unit *α*-*L*-glutamic acid. Owing to their biocompatibility, biodegradability, and non-immunogenicity, PGA-based nanomaterials have been elaborately designed for drug delivery systems. Relevant studies including the latest research results on PGA-based nanomaterials for drug delivery have been discussed in this work. The following related topics are summarized as: (1) a brief description of the synthetic strategies of PGAs; (2) an elaborated presentation of the evolving applications of PGA in the areas of drug delivery, including the rational design, precise fabrication, and biological evaluation; (3) a profound discussion on the further development of PGA-based nanomaterials in drug delivery. In summary, the unique structures and superior properties enables PGA-based nanomaterials to represent as an enormous potential in biomaterials-related drug delivery areas.

## 1. Introduction

Cancer is one of the leading causes of death in humans. Although chemotherapy has gained enormous achievements in cancer treatment, the non-specific drug distribution-induced systemic side effects of anticancer drugs are still a Gordian knot [1,2]. Unremitting efforts have been made in the development of novel therapeutic regimens for a satisfactory curative effect [3,4,5,6,7]. Nanomaterials based on the incorporation of small molecule cancer drugs into the biocompatible polymers have attracted considerable attention because they can precisely deliver drugs to sites of action [8,9,10,11].

Poly(*α*-*L*-glutamic acid) (PGA) is a kind of synthetic polypeptide containing the monomeric unit *α*-*L*-glutamic acid [12]. Owing to their inherent properties including biocompatibility, biodegradability, non-immunogenicity, and till date, PGA-based nanomaterials have been extensively applied in biomedical fields such as cancer therapy, wound healing, medical devices, bio-sensing, and tissue regeneration [13,14,15]. Traditionally, PGAs are chemically synthesized by the ring-opening polymerization (ROP) of the *γ*-protected *L*-glutamate *N*-carboxyanhydrides (LG NCAs) [16,17]. After post deprotection, the bare carboxyl groups in each repeat unit of PGAs provide the high functionality for the chemical conjugation of molecules [18]. On the other hand, the water-soluble PGA moieties can serve as the hydrophilic building blocks of amphiphilic polymeric nanocarriers to physically entrap the therapeutic agents [19]. The pK_a_ of the pendent carboxyl side chains of PGAs is around 4.5. PGAs are negatively charged under physiological conditions; however, the pendent carboxyl side chains are positively charged when subject to acidic microenvironments, such as extracellular tumors (pH 6.8) and endosomes (pH 5.5) [12,20]. In addition, the ionic alternation transforms PGA moieties from hydrophilic and random-coiled conformation into hydrophobic and *α*-helical conformation, facilitating the stimuli-release of preloading.

**Figure 1:**
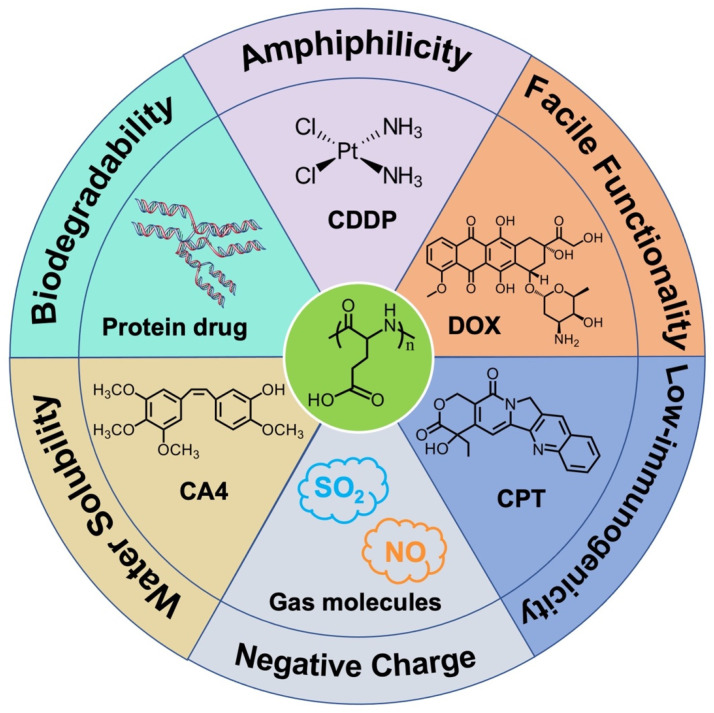
Schematic illustration of properties and the encapsulated drugs of PGA-based nanomaterials.

In this review, we highlight the recently vital achievements in the development of PGA-based nanomaterials for drug delivery (Figure 1). A brief description of the synthetic strategies of PGAs will be firstly summarized. Then, the evolving applications of PGA in drug delivery systems (DDS), including the rational design, precise fabrication, and biological evaluation, will be extensively discussed. The current challenges and future perspectives of PGA-based nanomaterials are also presented.

## 2. Synthesis of PGA

Synthetic polypeptides are generally prepared from three methods: solid-phase peptide synthesis (SPPS), NCA polymerization, and microbial fermentation [12,20]. SPPS enables the automated preparation of the defined sequenced polypeptides via convenient isolation and purifications steps but is restricted to low molecular weight polymers (typically lower than 50 m) [21,22]. Microbial fermentation is an effective protocol to prepare high molecular weight and sequenced polypeptides, yet specialized and sophisticated equipment is not feasible for most synthetic laboratories [23,24,25]. NCA polymerization has been recognized as an economical and expedient technique that allows the fabrication of polypeptides with high yields, predetermined composition, narrow molecular weight distribution, and tuned functionality [26,27]. Furthermore, it’s a versatile and scalable method to synthesize polypeptides and is suitable for diverse types of NCAs. The past ten years has seen considerable developments in the polymerization of NCAs. This section will highlight the important advances on the chemical synthesis of PGAs.

**Scheme 1:**
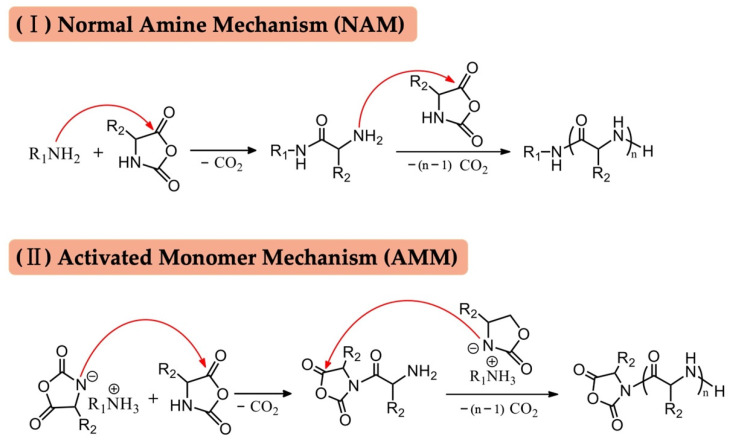
Two widely accepted mechanisms of amine–initiated NCA polymerization.

Chemical synthesis of PGAs involves ROP of LG NCAs. Benzyl and tert-butyl are the most frequently used groups to protect the *γ*-carboxylic acid of *L*-glutamic acid because they are readily removable [28,29]. ROP of NCAs is generally triggered by the protic/nonprotic nucleophile or strong/weak base. The amine-initiated ROP of LG NCAs is the most prevalently used method to synthesis PGAs. There are two widely accepted mechanisms, the “normal amine mechanism” (NAM) and the “activated monomer mechanism” (AMM), which occur simultaneously and complicate the polymerization process (Scheme 1) [30,31]. NAM typically initiates by a primary amine and presents a slow but controlled polymerization, whereas AMM generally mediates by a tertiary amine and gives a fast but uncontrolled polymerization.

**Scheme 2:**
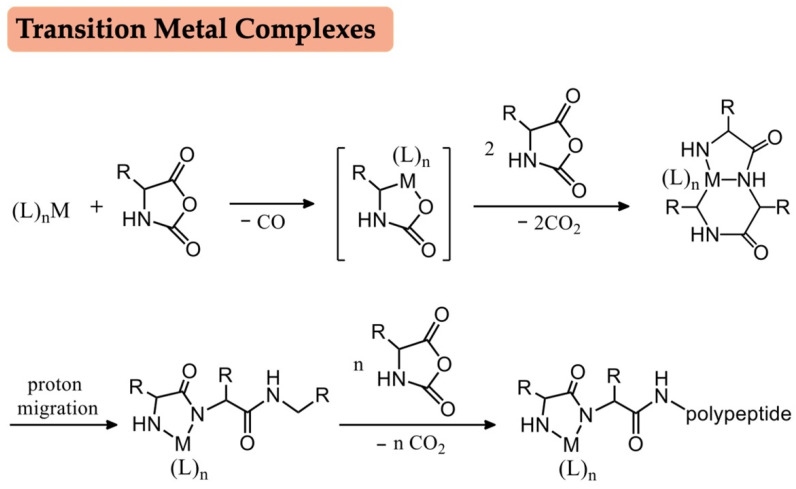
Transition metal complexes initiated NCA polymerization.

**Scheme 3:**
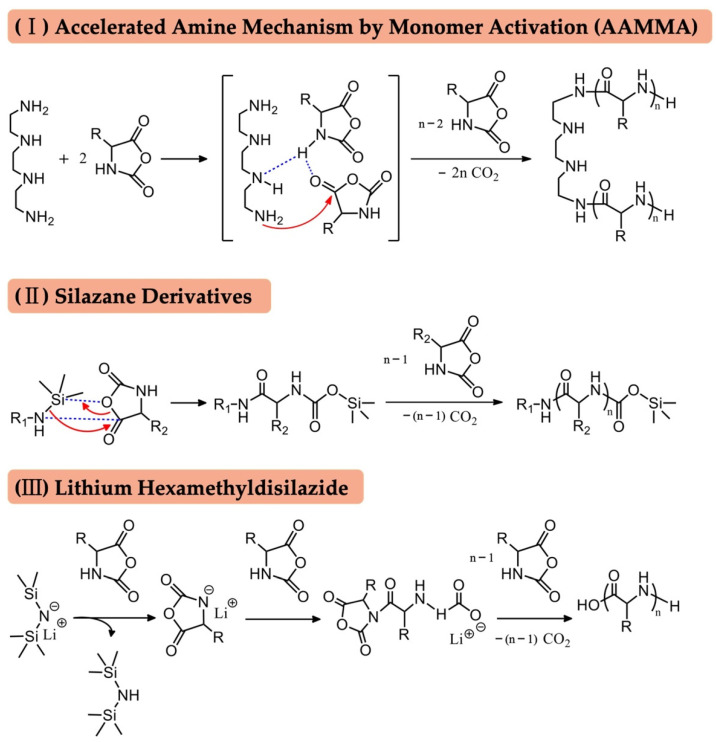
Novel NCA polymerization methods based on amine initiators.

Recent decades have witnessed great strides in the polymerization of NCAs, and various new initiators and catalysts have been reported for mediating controlled NCA polymerization. Transition metal complexes pioneered by Deming have emerged as efficient initiators to obtain a narrow molecular weight distribution, yet the inevitable removal of the metallic residues have restricted the general application of metal initiators (Scheme 2) [32,33]. Considering the drawbacks of NAM and AMM, Hadjichristidis et al. proposed a peculiar mechanism, the “accelerated amine mechanism by monomer activation” mechanism (AAMMA), based on an “alliance” of primary and secondary amine initiators, including triethylaminetriamine, hexamethyldiamine and *N*,*N*′-dimethyl-1,2-ethanediamine (Scheme 3) [34,35]. Silazane derivatives provide a possibility for the controlled NCA polymerization. As reported by Lu, hexamethyldisilazane, trimethylsilyl (TMS) and their derivatives could polymerize *γ*-benzyl-*L*-glutamate NCAs (BLG NCAs) in a well-controlled manner (Scheme 3) [36,37]. Moreover, the Cheng group recently developed an auto-accelerated polymerization of *α*-helical polypeptides based on the TMS protected amine groups, where the synergistic interaction of macrodipoles between neighboring *α*-helices of polypeptides remarkably accelerate the ROP of NCAs [38]. The cooperative interaction involving *α*-helical of polypeptides also has been extended to diamine-initiated ROP of NCAs [39]. It is well known that NCA polymerization is significantly sensitive to humid environments and must be carried out under an inert atmosphere. Surprisingly, Liu et al. demonstrated that lithium hexamethyldisilazide can offer an extremely rapid ROP of NCAs either in the glovebox or in open vessel conditions (Scheme 3) [40].

**Scheme 4:**
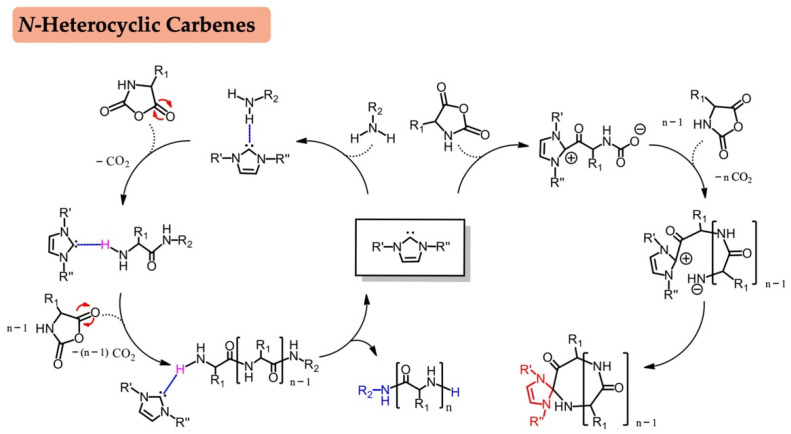
NHC–mediated NCA polymerization to access linear and cyclic polypeptides.

**Scheme 5:**
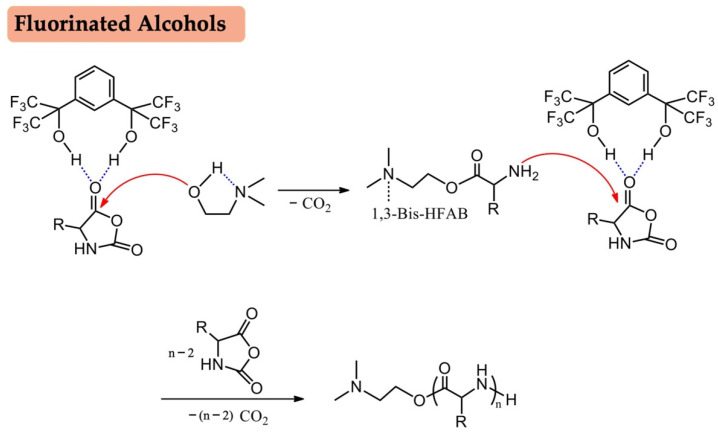
Fluorinated alcohol–initiated NCA polymerization.

Hydrogen bonding interaction is a potent weapon to achieve controllable NCA polymerization. Our lab firstly discovered that imidazolium hydrogen carbonate salts, the precatalysts of *N*-Heterocyclic carbenes (NHC), can hydrogen bond with primary amine initiators as well as ω-terminus of the propagated polypeptide chain, generating linear structured polypeptides, whereas cyclic polypeptides are obtained in the absence of the primary amine initiators (Scheme 4) [41,42]. Compared to amine initiators, the less nucleophilic alcohols usually lead to a slow initiation and poorly controlled polypeptides. Continuous efforts have been devoted to break the ice and enhance the nucleophilicity of alcohol. Based on the massive experiments and rigorous reasoning, it was reported that hydrogen bonding interactions with diazabicycloundecene, triazabycyclodecene, thioureas, and fluorinated alcohols can synchronously improve the nucleophilicity of the alcohol initiators, propagate hydroxyl terminus and activate the NCA monomers (Scheme 5) [43,44,45].

Emulsification offers an alternative to traditionally amine initiated-NCA polymerization [46]. More recently, Cheng et al. have reported a water-in-oil emulsion to polymerize BLG NCAs by using a macroinitiator, affording an accelerating polymerization rate and the well-controlled polypeptide extension [47]. The concepts of frustrated Lewis pairs and self-assembly have also been explored to control the ROP of NCAs [48,49]. Aimed to achieve the controlled NCA polymerization, reaction conditions such as vacuum, temperature, photo, nitrogen flow, and solvent have also been carefully designed and optimized, except in developing emerging initiators and catalysts [50,51,52]. Given the impressive achievement in the preparation of polypeptides, diverse polypeptides-derived nanomaterials with predictable molecular weights, low dispersity, and defined functionalities can be constructed. Nevertheless, there is a huge gap between the synthetic polypeptides-derived nanomaterials and the clinically used one. In addition, the property and function of synthetic polypeptides are still far from matching the natural proteins. Further efforts in chemical synthesis of polypeptides with controllable monomer sequence, anticipated structure, and protein-like functions are required to achieve the preparation of clinically feasible biomaterials.

## 3. PGA-Based Nanomaterials for Drug Delivery

Chemotherapy is one of the crucial tumor therapeutic regimens in clinic [53,54,55,56]. To improve therapeutic efficacy and minimize nonspecific toxicity, the development of DDS that could enable the controllable release of chemical drug, alleviate premature drug leakage, and target specific tissues remains an enormous challenge [57,58,59,60]. PGA-based nanomaterials have gained increasing popularity in DDS due to their defined structure, tuned functionality, superior biocompatibility, and low immuneogenicity [61,62,63,64]. It is reported that PGA-based nanocarriers can specifically adhere to the *β*-glutamyl transpeptidase at the tumor cell membranes and overcome the serum inhibitory effect [65,66,67]. Various of chemotherapeutic drugs, such as doxorubicin (DOX), camptothecin (CPT), vascular disrupting agents (VDA), and platinum (Pt) drugs, have been chemically conjugated or physically encapsulated to PGA-based nanomaterials (Table 1) [68,69,70,71,72,73,74,75,76,77,78,79,80,81,82,83,84,85,86,87].

### 3.1. PGA-Based Nanomaterials as DOX Delivery Systems

**Figure 2:**
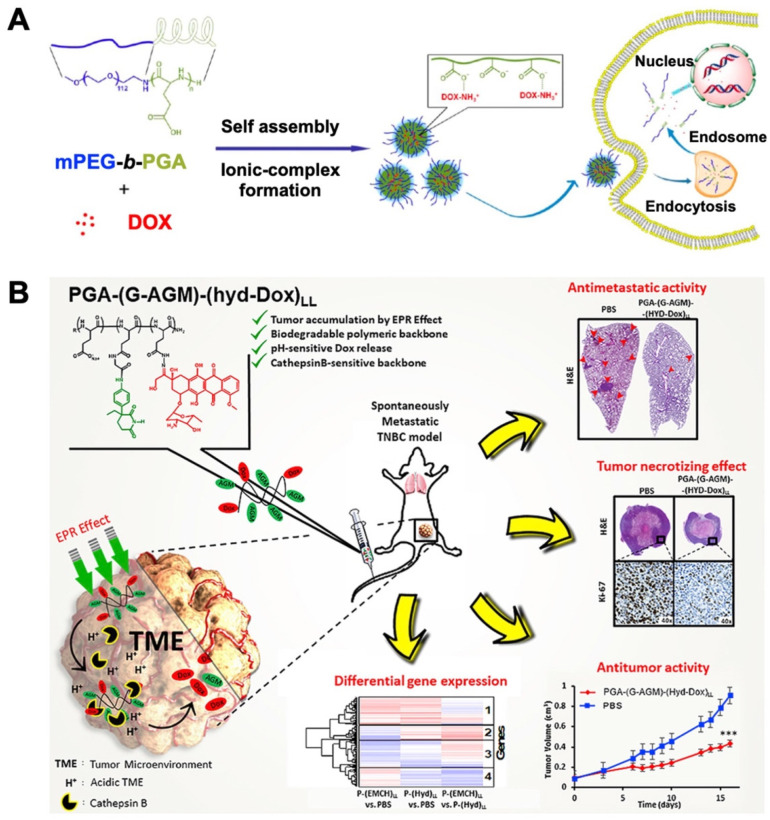
Fabrication of PGA-based nanomaterials by chemical conjugation and physical encapsulation of DOX. (**A**) Schematic illustration of the preparation and endocytosis mechanism of the pH–responsive mPEG–*b*–PGA amphiphiles. Reprinted with permission from Ref. [71]. Copyright 2013, American Chemical Society. (**B**) Synthetic procedures of PGA–based combination conjugates, *** *p* < 0.001. Reprinted with permission from Ref. [73]. Copyright 2018, Elsevier.

DOX, an anthracycline antibiotic, is the most prevalently used chemotherapeutic drug for the treatment of various cancers, such as cancers of the breast, stomach, lung, thyroid, ovary, and bladder [88,89,90]. DOX can penetrate the endonuclear DNA and suppress DNA replication, leading to cell apoptosis. Nevertheless, the short blood circulation period and inevitable adverse effects including cardiotoxicity, nephrotoxicity, and myelosuppression significantly reduce its therapeutic outcomes [91,92]. Chemical conjugation and physical encapsulation of DOX to biodegradable PGA present a viable method to enhance its bioavailability and reduce side effects [93,94,95,96,97,98]. Traditionally, hydrophobic drug loading procedures contain the dissolution of the amphiphilic polymers and small molecular drugs in organic solvents, and the subsequent removal of the organic solvents by solvent evaporation or dialysis [99]. Unlike conventional encapsulation procedures, PGA-based anionic polymers are specifically complex, with cationic DOX through electrostatic interactions, omitting the use of detrimental organic solvents and achieving approximately 100% loading efficacy [100]. Chen et al. designed a poly(ethylene glycol)-*b*-poly(*α*-*L*-glutamic acid) (mPEG-*b*-PGA) nanocarrier entrapped with DOX through electrostatic interaction and an intermolecular hydrophobic stack (Figure 2A) [71]. The PEG segments were mainly oriented at the outer periphery of nanocarriers preventing the adsorption of protein and identification by the phagocyte system, whereas the PGA segments were primarily situated in the aqueous interior of nanocarriers warranting high loading of DOX. Cellular uptake tests suggested that the resultant nanocarriers were up-taken into A549 cells via endocytosis. Subsequently, the endosomal acidic condition triggered the destabilization of nanocarriers, resulting in the release of DOX to cytoplasm. Because of the enhanced permeability and retention (EPR) effect, the resultant ionomer complex exhibited a prolonged blood circulation period, decreased systemic toxicity, and enhanced therapeutic efficacy in the treatment of nonsmall cell lung cancer. To enhance the stability of PGA-based nanocarriers, hydrophobic units such as leucine (Leu) and phenylalanine (Phe) are incorporated to construct three monomeric units of the copolypeptides [100,101].

Pioneered by Kataoka, who firstly conjugated DOX to the pendent carboxyl acids of poly(ethylene glycol)-*b*-poly(*L*-aspartate) (mPEG-*b*-PLA), chemical conjugation of DOX onto polypeptides also have attracted considerable attention in cancer therapy [102,103,104,105,106]. Xiao et al. developed a pH and redox dual-stimuli poly(ethylene glycol)-*b*-poly(*γ*-propargyl-*L*-glutamate) (mPEG-*b*-PPLG) prodrug nanogel by simultaneously coordinating DOX through an acid-labile hydrazone bond and cross-linking with a redox sensitive 2-azidoethyl disulfide bond via one-step “click chemistry” [72]. The resultant mPEG-*b*-PPLG prodrug nanogels exhibited elevated stability during blood circulation and stimuli release of DOX in tumor cells. Recently, Vicent also developed a family of PGA-based combination conjugates bearing chemotherapeutic drug (DOX) and aromatase inhibitors (aminoglutethimide, AGM) for the treatment of breast cancer (Figure 2B) [73,107]. DOX was directly bound to the carboxyl groups of PGA either by amide bond or acid-labile hydrazone bond, whereas AGM was incorporated into PGA via a library of glycine (Gly) linkages (such as Gly linker, Gly–Gly linker, and Gly-Phe-Leu-Gly linker), which are readily cleaved by protease Cathepsin B. The controllable release of DOX and AGM in intracellular microenvironments enabled the superior therapeutic effects on primary tumor growth, apoptosis of cancer cells, and lung metastasis. PGA-based biomaterials provide a superior nanoplatform for small molecular drugs and achieved an enhanced therapeutic effect in tumor therapy. These pioneering examples pave the way for chemical conjugation and physical encapsulation of chemotherapeutics by using PGA-based nanomaterials.

### 3.2. PGA-Based Nanomaterials as Pt Drugs Delivery Systems

Hydrophobic Pt drugs like cisplatin (CDDP), carboplatin, and oxaliplatin have become promising candidates for the treatment of malignant tumors [108,109,110]. Pt drugs can contact DNA to disrupt its replication and eventually result in the apoptosis of tumor cells [111], whereas extremely low solubility and severe side effects significantly reduce its tumor therapeutic efficacy [112,113]. To overcome this restriction, a variety of polymeric nanocarriers have been explored to entrap Pt drugs [114,115]. Kataoka et al. firstly attempted to conjugate CDDP to the pendent carboxyl groups of mPEG-*b*-PLA [116]. To note, a series of Pt drugs coordinated mPEG-*b*-PGA, such as NC-6004 and NC-4016, have been assessed in phase III clinical trials for patients with advanced or metastatic pancreatic cancer [117,118]. The hydrophilic shell endows both NC-6004 and NC-4016 with a long blood circulation period and increased drug accumulation in the targeted tumor tissues through EPR effect.

**Figure 3:**
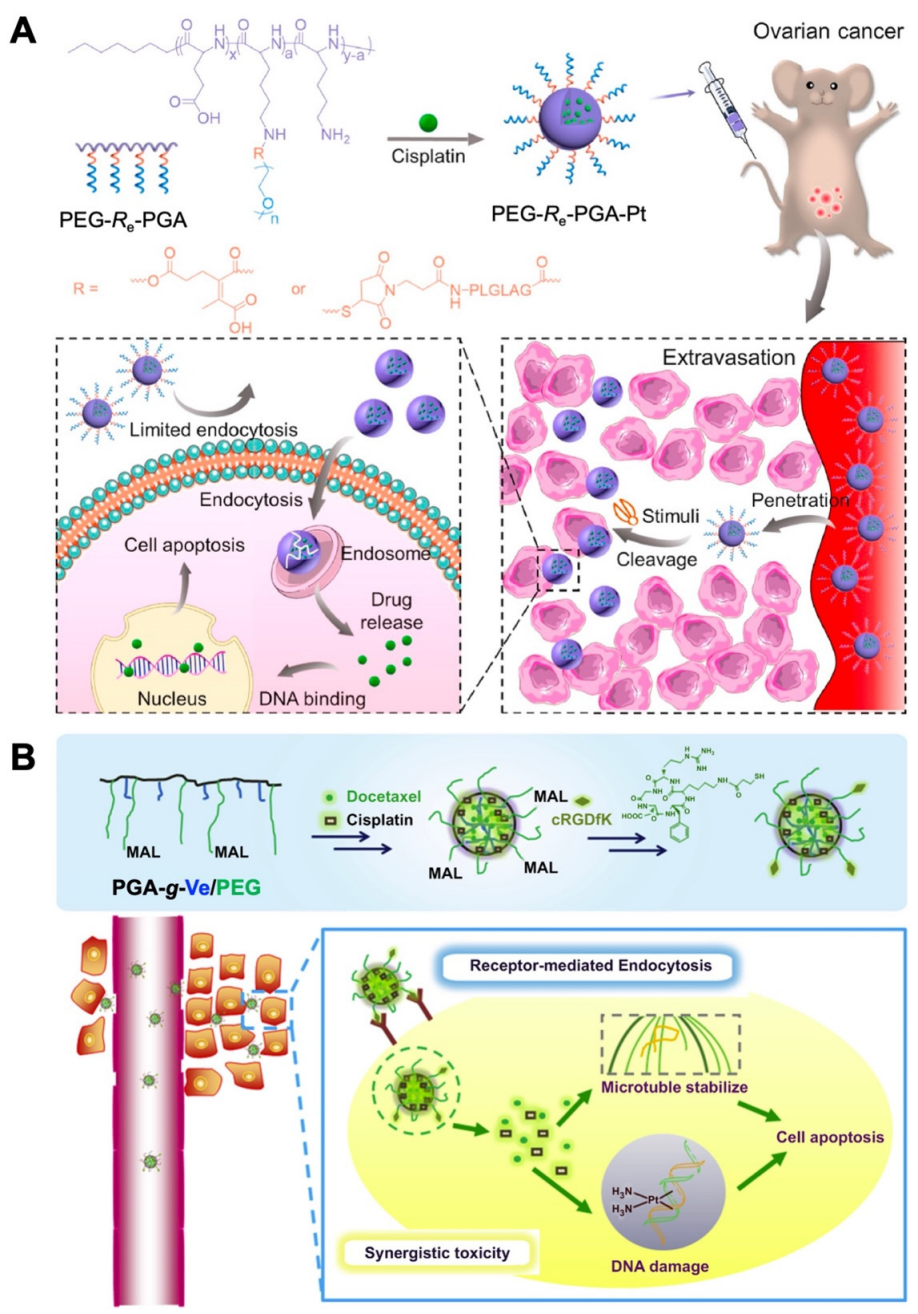
Pt drugs were encapsulated in PGA-based nanomaterials for the treatment of cancer. (**A**) Schematic illustration of PGA-Pt nanocarriers with cleavable PEG response to tumor microenvironments. Reprinted with permission from Ref. [74]. Copyright 2021, Elsevier. (**B**) DTX/CDDP co-encapsulated nanocarriers decorated with the targeting of c(RGDfK) enter tumor tissues by receptor-mediated cellular uptake. Reprinted with permission from Ref. [75]. Copyright 2013, Elsevier.

Even so, the resistance and internalization dilemmas like free cisplatin still exist. A major reason lies in the steric repulsion of the dense PEG shell, which inevitably results in the PEGylated Pt drugs-conjugated nanocarriers bypassing the tumor tissues or failing to be phagocytosed by the tumor tissues [119]. To overcome these obstacles, chemical and physical dePEGylation induced by particular tumor microenvironments, such as pH, redox, and enzyme, have been deeply explored [120,121]. More recently, Xu et al. fabricated two types of poly(*L*-glutamic acid)-cisplatin (PGA-Pt) nanocarriers with cleavable PEG, which are sensitive to extracellular pH (pH_e_) and matrix metalloproteinases-2/9 (MMP-2/9) [74]. As displayed in Figure 3A, the pH_e_-sensitive 2-propionic-3-methylmaleic anhydride (CDM)-derived amide linkage and MMP-2/9-responsive cleaved peptide PLGLAG were designed to bridge PGA and PEG, generating pH_e_-sensitive PEG-pH_e_-PGA and MMP-2/9-sensitive PEG-MMP-PGA. CDDP was coordinated with the corresponding graft copolymers, yielding the polymer–metal complexed nanoplatforms, PEG-pH_e_-PGA-Pt and PEG-MMP-PGA-Pt. Cellular uptake assays revealed that PEG-PGA-Pt exhibited the limited cell internalization in SKOV3 cells due to the steric repulsion between the dense PEG shell and cell membrane. Conversely, PEG-pH_e_-PGA-Pt exhibited a significantly higher cell internalization in SKOV3 cells due to the dePEGylation triggered by the cleavage of the CDM-derived amide bond. The endosomal pH condition induced the instability of the bare PGA-Pt core, leading to the increased release of CDDP into cytol. Compared to the traditional PEG-PGA-Pt, the detachable PEG-pH_e_-PGA-Pt and PEG-MMP-PGA-Pt not only retained the prolonged circulation time, the pH and MMP detachable PEGylated PGA-Pt nanoformulations enabled the enhanced cell internalization toward the high-grade serous ovarian cancer, eventually leading to the up-regulated antitumor efficacy.

Tang et al. integrated the merits of the “receptor-mediated cellular uptake” and “multi-drug delivery” into one nanoformulation (Figure 3B) [75]. Docetaxel (DTX) and CDDP were co-encapsulated into the amphiphilic poly(*L*-glutamic acid)-*g*-*α*-tocopherol/polyethylene glycol (PGA-*g*-Ve/PEG) nanocarriers through hydrophobic and chelation interaction, followed by the periphery decoration of an avb3 integrin targeting peptide c(RGDfK). Thanks to the targeting c(RGDfK), DTX/CDDP co-encapsulated nanoformulation exhibited a synergistically increased accumulation rate and retention time in mouse melanoma cells. Folic acid (FA), an active targeting ligand, has also been extensively utilized for targeted CDDP delivery. Qiao et al. recently designed the CDDP-loaded maleimide-poly(ethylene oxide)_114_-*b*-poly(*L*-glutamic acid)_12_ (Mal-PEG_114_-*b*-PLG_12_) vesicles for the targeted delivery of CDDP to tumor sites [76]. CDDP complexed to PGA moieties induced the self-assembly of the copolymer into vesicular morphologies via the formation of a hydrophobic domain, while PEG blocks served as the corona and interior layer of the vesicular morphologies. The reactive maleimide groups on the vesicle periphery could conjugate with FA thiol, yielding an active targeted DDS, which presented distinctly high cellular uptake and desired cytotoxicity toward HeLa cells. Targeting agents enable the targeted CDDP delivery to tumor sites, yet the dedicated and complicated modification procedures also increase the potential system toxicity.

### 3.3. PGA-Based Nanomaterials as CPT Delivery Systems

**Figure 4:**
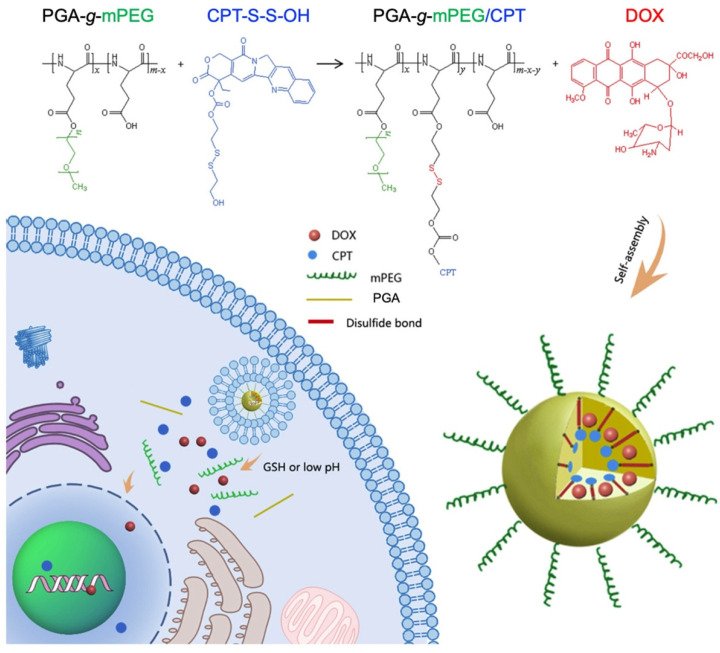
Construction of PGA–*g*–mPEG-based CPT conjugate and its encapsulation of DOX for GSH-triggered dual drug release in cancer cells. Reprinted with permission from Ref. [77]. Copyright 2018, Elsevier.

As a topoisomerase I inhibitor, CPT which is derived from the Chinese tree Camptotheca acuminata, can induce a variety of tumor cell apoptosis [122,123,124,125]. Unfortunately, the relatively low aqueous solubility and pH-dependent lactone ring stability of CPT severely constrain its clinical application [126]. Towards this end, both Singer and Klein’s groups demonstrated that the water solubility and lactone ring stability could greatly be enhanced by the conjugation of CPT to the residing carboxylic acid of PGA [127,128]. Researchers have also combined CPT with other chemotherapeutic drugs for synergistic cancer treatment [77,129]. Xiao et al. developed a redox responsive nanoformulation via the self-assembly of poly(*L*-glutamic acid)-*g*-poly(ethylene oxide) (PGA-*g*-mPEG) based CPT conjugate and simultaneous entrapment of DOX by hydrophobic interaction (Figure 4) [77]. CPT was linked to PGA-*g*-mPEG via a disulfide bridge which was readily detachable in a glutathione (GSH) environment. It was observed that the intracellular GSH concentration plays a decisive role in the release of DOX and CPT from nanoformulation. As proved by the flow cytometry and the cellular uptake tests, the acidic endo-lysosomal microenvironment induced the release of DOX, GSH in the cytoplasm, and cleaved the disulfides, releasing CPT from the resultant nanoformulation. The low combination index value (approximately ~0.3) substantiated the valid cancer cell apoptosis based on the nanoformulation co-loaded with CPT and DOX. A semisynthetic analog of CPT, 7-Ethyl-10-hydroxy camptothecin (SN38), has been approved by FDA for colorectal carcinoma therapy [130]. Recently, Tamaddon et al. prepared a double hydrophilic poly(2-ethyl 2-oxazoline) block poly (*L*-glutamic acid) (PEtOx-*b*-PGA) prodrug by coupling SN38 to the pendent carboxyl group of PGA [78]. Compared to free drugs, cell culture assays displayed a higher intracellular accumulation and at least four times more specific cytotoxicity than the coupled SN38 in the CT26 cell line. Moreover, the as-prepared SN38 conjugate exhibited outstanding anti-tumor activity and was significantly superior to commercial irinotecan, especially on advanced tumors with a reduced mortality rate of 2.5 times. All these studies suggest that CPT-derived topoisomerase I inhibitors can exert their tumor cell apoptosis effect in tumor therapy. Further efforts are needed to investigate the more detailed action mechanism of these nanoformulations.

### 3.4. PGA-Based Nanomaterials as VDA Delivery Systems

**Figure 5:**
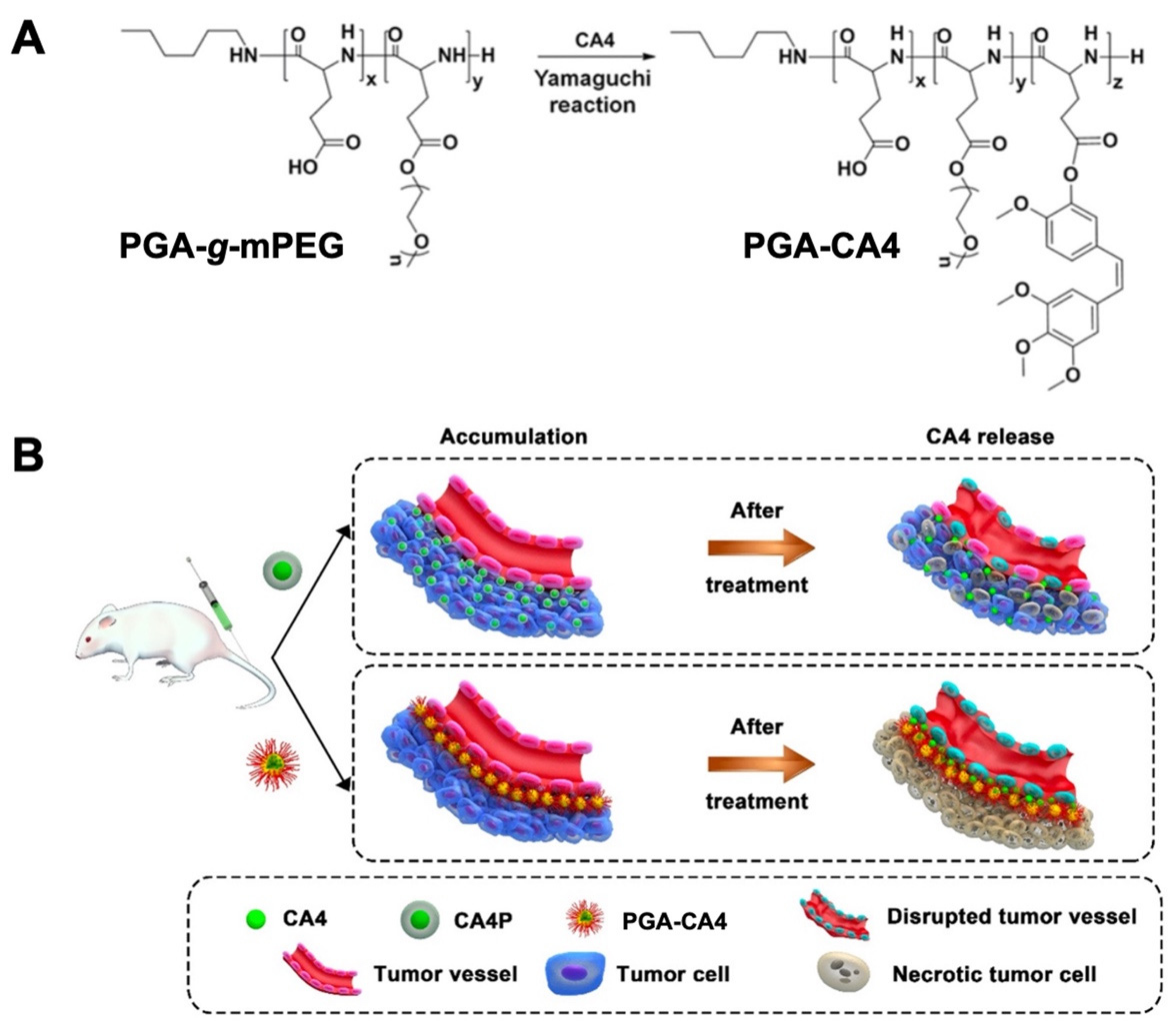
Comparation of the mechanism of PGA–CA4 and CA4P for the treatment of the murine colon C26 tumor cell. (**A**) Preparation of PGA–CA4 conjugate. (**B**) Mechanistic illustration of PGA-CA4 and CA4P toward tumor cells. Reproduced with permission. Reprinted with permission from Ref. [79]. Copyright 2017, Elsevier.

VDA can selectively modulate tumor vasculature and rapidly induce the shutdown of tumor blood vessels, leading to widespread tumor cell ischemic necrosis [131,132]. Combretastatin A4 (CA4) is a crucial agent for clinical cancer therapy. As a kind of microtubule depolymerizing agent, CA4 can attach to the colchicine adhering site of *β*-tubulin, resulting in cytoskeletal destabilization and morphological variation of the endothelial cell [133,134,135]. The poor aqueous solubility of CA4 is the greatest hindrance for the extensively clinical application. Tong et al. designed a polymeric CA4 conjugate via coordination of CA4 to poly(*L*-glutamic acid)-CA4 (PGA-CA4) (Figure 5A) [79]. Intra-tumor distribution experiments indicated that PGA-CA4 nanoconjugates were predominantly localized around tumor blood vessels due to the active targeting property of CA4. This enabled the long-term release of CA4 inside solid tumor cells. The gradually increased CA4 concentration around tumor blood vessels caused the steady tumor blood deprivation and effective tumor regression (Figure 5B). Owing to the vascular-dependent distribution character, the obtained PGA-CA4 exhibited a long retention time in plasma and the murine colon C26 tumor cell compared to commercial combretastatin-A4 phosphate (CA4P). After a single administration, PGA-CA4 induced enduring angiorrhexis and tumor destruction in 72 h, leading to a tumor suppression rate of 74%.

**Figure 6:**
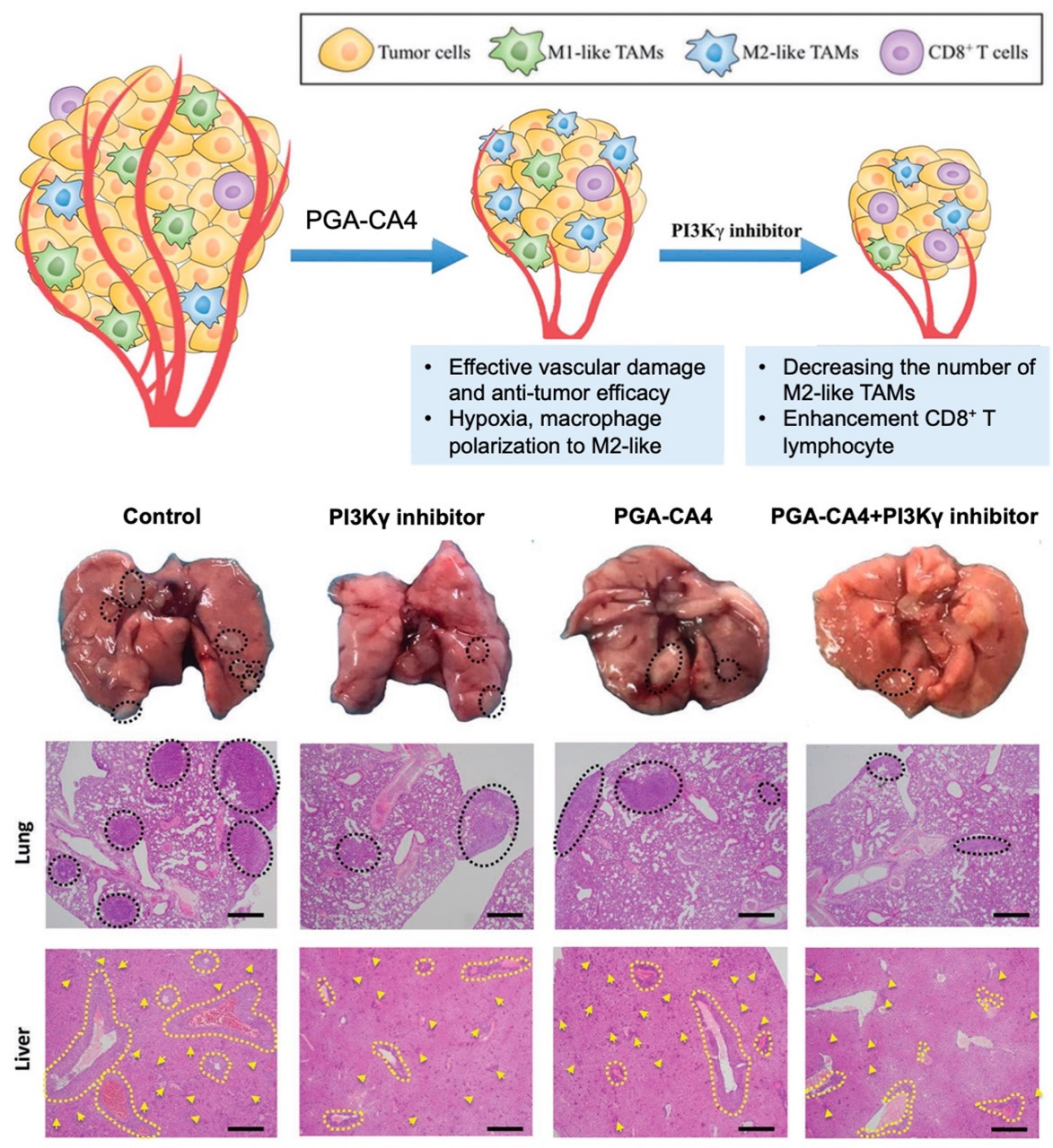
Reduction of the breast cancer metastasis based on PGA-CA4 in synergic with PI3K*γ* inhibitor. Optical photographs of lungs and H&E images of lung and liver for control and mono-/dual-prodrug treated groups. Scale bar = 500 µm. Reprinted with permission from Ref. [80]. Copyright 2019, Wiley-VCH GmbH, updated by CC 4.0.

However, several side effects, such as polarization induced by PGA-CA4, significantly restricted the antitumor activity. To this end, the same group also combined this nanomedicine with other antineoplastic agents for enhanced cancer therapy [136,137]. For instance, they utilized the phosphoinositide 3-kinase gamma isoform (PI3K*γ*) selective inhibitors synergizing with PGA-CA4 to reduce the immunosuppressive effects (Figure 6) [80]. The number of M2-like tumor-related macrophage obviously decreased while the cytotoxic T lymphocytes markedly improved due to PI3K*γ*inhibitor. Remarkably, the combination of PI3K*γ* inhibitor and PGA-CA4 prevented the tumor growth and extended the mean survival time, significantly enhancing the tumor therapeutic efficacy (Figure 6). Even though CA4-conjugated nanocarriers effectively inhibited tumor growth and tumor proliferation, CA4 could not be tested by multispectral optoacoustic tomography and immunofluorescence assay, hindering the observation of the intra-tumor distribution of CA4.

### 3.5. PGA-Based Nanomaterials as Gas Molecule Delivery Systems

Mammalian tissues generate many kinds of gas molecules, such as nitric oxide (NO), carbon monoxide (CO), and sulfur dioxide (SO_2_), which play a transmitter role in a series of biological activities and regulate the biochemical or physiological processes in the human body [138]. Recently, gas therapy has become an emerging tumor therapeutic technique because there is no drug resistance, it has minimal side effects and there is no byproduct [139]. Numerous gas nanogenerators are designed to delivery and produce safe gas molecules for the treatment of tumors [140,141]. NO, is an endogenously generated radical gas molecule, involved in various physiological functions, such as cardiovascular homeostasis, neurotransmission, and immune response to infection and angiogenesis [142]. NO can modulate P-glycoprotein expression without multi-drug resistance at low dosages, while high concentrations of NO can damage DNA and mitochondria in solid tumors, leading to cell mortality [143]. Hong et al. fabricated (poly-*L*-lysine/poly-*L*-glutamic acid)_n_ (PLL/PGA)_n_ multilayer films with different thicknesses for controlled NO releasing [81]. By applying the layer-by-layer self-assembly approach, PLL and PGA were employed to construct the multilayer films via electrostatic interaction, where PLL served as the positively charged blocks and PGA acted as the negatively charged blocks. A proton-responsive NO donor, *N*-diazeniumdiolate, was loaded into (PLL/PGA)_n_ multilayer films via a high pressure reaction under NO atmosphere. The as-obtained (PLL/PGA)_n_ multilayer films displayed a continued NO releasing behavior, suggesting the controllable NO delivery for tumor treatment. SO_2_ has been recognized as a promising gasotransmitter for regulation of the cardiovascular system. Xiao et al. developed a polymeric GSH-responsive nanomedicine of SO_2_ to combat MCF-7 ADR human breast cancer cells in synergy with DOX [82,144]. *N*-(3-azidopropyl)-2,4-dinitrobenzenesulfonamide (AP-DNs), a small molecular generator of SO_2_, was coupled onto the pendent groups of methoxy poly(ethylene glycol)-*block*-poly (*g*-propargyl-*L*-glutamate) (mPEG-*b*-PPLG) copolymer via “click chemistry”, yielding the polymeric nanomedicine of SO_2_, mPEG-*b*-PLG (DNs). DOX was finally encapsulated into mPEG-*b*-PLG (DNs) nanomedicine via self-assembly. Upon GSH triggering, the obtained mPEG-*b*-PLG (DNs) nanomedicine simultaneously released SO_2_ and DOX, causing an enhancement of reactive oxygen species (ROS) in tumor tissue and synergistic anti-proliferation effects against MCF-7/ADR cells.

### 3.6. PGA-Based Nanomaterials as Co-Delivery Systems

Co-delivery of dual antineoplastic agents in a polymeric nanocarrier has attracted enormous interest due to the synergistic therapeutic effect [145,146,147,148,149]. Tremendous efforts have been devoted to exploring the combined treatment based on chemotherapy, phototherapy, biological therapy, and radiation therapy. Photodynamic therapy (PDT) is a kind of phototherapy involving light irradiation and photosensitizer. Upon being activated by specific light irradiation, photosensitizer can produce ROS to induce the tumor cell apoptosis [150]. Yu et al. designed CA4 and porphyrin (5, 10, 15, 20-tetraphenylporphyrin, TPP)-conjugated nanomedicines, CA4-conjugated poly(*L*-glutamic acid)-*graft*-methoxy poly(ethylene glycol) (PGA-*g*-mPEG-CA4), and TPP-conjugated PGA-*g*-mPEG (PGA-*g*-mPEG-TPP), for combined vascular disrupting photodynamic therapy [83,151]. Upon laser irradiation, the PGA-*g*-mPEG-CA4 nanomedicines exhibited superior antitumor ability and PGA-*g*-mPEG-TPP nanomedicines generated dioxygen to kill cancer cells. However, the combination of PGA-*g*-mPEG-CA4 and PGA-*g*-mPEG-TPP nanomedicines connected the effect of vascular blockage and photodynamic cell apoptosis, attaining the efficacy of interior and exterior tumor cell killing.

**Figure 7:**
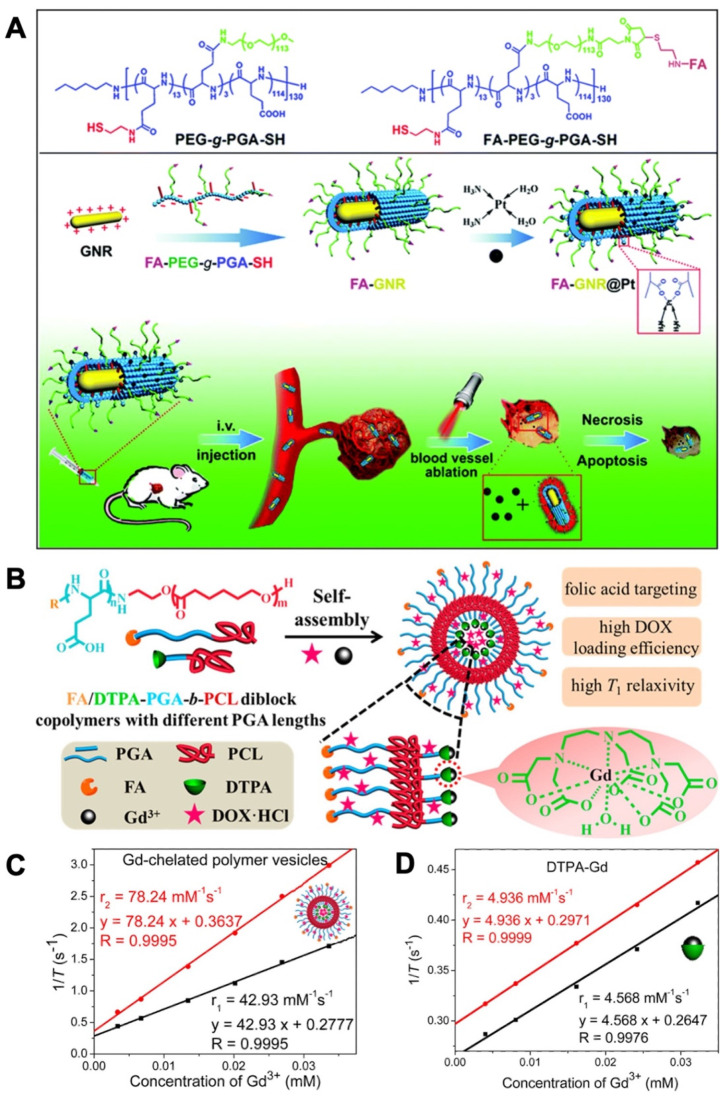
Combined treatment based on chemotherapy, PTT, and MRI. (**A**) Combinational chemo-photothermal therapy of breast cancer by utilizing the FA–targeted GNR@Pt prodrug. Reprinted with permission from Ref. [84]. Copyright 2015, The Royal Society of Chemistry. (**B**) Asymmetrical polymeric vesicles with multi functions for ultrasensitive MRI and targeted tumor therapy. (**C**,**D**) The T1 relaxivity of Gd(III) –chelated asymmetrical vesicles and traditional DTPA–Gd. Reproduced with permission. Reprinted with permission from Ref. [85]. Copyright 2015, American Chemical Society.

Photothermal therapy (PTT), another type of phototherapy, can result in apoptosis or necrocytosis of the tumor issues and inhibit tumor proliferation by inducing the partial hyperthermia effect of photothermal agents [152]. Li et al. constructed gold nanorods (GNRs) with CDDP-methoxy poly(ethylene glycol)-*graft*-poly(*L*-glutamic acid) (CDDP-mPEG-*g*-PGA) wrapping and FA decoration (FA-GNR@Pt) for the targeting of chemo-photothermal therapy of breast cancer (Figure 7A) [84]. The chemical conjugation of GNRs to mPEG-*g*-PGA copolymers with thiol groups could efficiently remove the cetyl trimethylammonium bromide moieties and minimize the toxicity of GNRs. To avoid protein absorption and prolong blood circulation, CDDP was complexed into the inner PGA core and FA were linked to the outer PEG corona. The as-prepared FA-GNR@Pt prodrug significantly suppress the growth and lung metastasis of the 4T1 breast tumor due to FA- mediated tumor targeting effects, and CDDP caused cellular apoptosis in synergy with near infrared laser illumination-induced cellular necrosis and ablation of the peripheral blood vessels.

Owing to easy operation and deep penetration into soft tissues, magnetic resonance imaging (MRI)-based techniques have been extensively applied in clinic treatment. Conventional T1-type contrast agents are usually restricted because of the risk of accumulated toxicity because of poor sensitivity. Du et al. proposed a noncytotoxic targeting polymer vesicle based on FA or diethylenetriaminepentacetatic acid (DTPA) poly(*L*-glutamic acid)-*b*-poly(*ε*-caprolactone) (FA/DTPA-PGA-*b*-PCL) (Figure 7B) [85]. The DOX and Gd(III) co-encapsulated asymmetrical polymer vesicles were obtained via the self-assembly of FA-PGA-*b*-PCL copolymers, where the hydrophobic PCL blocks act as the vesicular membranes and the hydrophilic PGA blocks serve as the vesicular coronas. Among which, the longer PGA blocks linked with FA contributed as the external coronas, whereas the shorter PGA blocks attached with DTPA acted as the internal coronas. Compared to traditional DTPA-Gd, such asymmetrical vesicles presented a high T1 relaxivity of 42.93 mM^−1^·s^−1^ and drug loading efficiency of 52.6% for DOX·HCl (Figure 7C,D). Moreover, in vivo MR imaging assays suggested an evident enhancement of the signal intensity around the targeted tumor sites. Compared to chemotherapeutics-based monotherapy, these dual therapy systems significantly improved the therapeutic effect in a synergistic way. New approaches with more simple, efficient, and economical properties are desired to fabricate the dual therapeutic nanoplatforms.

### 3.7. PGA-Based Nanomaterials as Protein Delivery Systems

**Figure 8:**
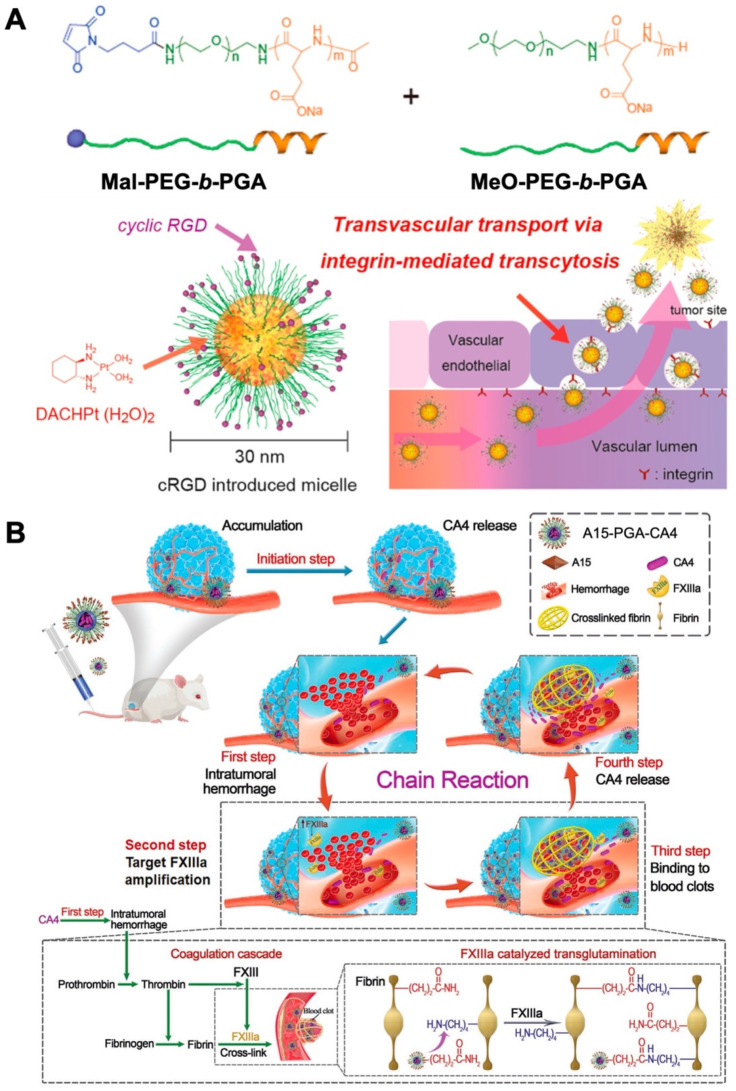
Protein drug-based PGA prodrugs for cancer treatment. (**A**) Preparation of DACHPt-incorporating PEG-*b*-PGA polymeric micelle with cRGD ligand molecules for the targeted delivery of platinum therapeutic prodrugs to glioblastomas. Reprinted with permission from Ref. [86]. Copyright 2013, American Chemical Society. (**B**) Schematic illustration of the self-amplifying tumor-homing therapeutic nanoplatform working via a chain reaction mechanism. Reprinted with permission from Ref. [87]. Copyright 2020, Wiley-VCH GmbH.

Since the approval of the first protein drug Humulin^®^ in 1982, hundreds of therapeutic protein drugs have been approved [153,154]. Peptide-based therapeutics have gained great momentum, owing to their high specificity and efficiency, fewer side effects, and that they are tolerated well by the human body [155,156]. Unfortunately, the peculiar hierarchical architectures of peptide drugs also endow them with inherent pharmaceutical defects, including poor stability, immunogenicity, and shorter retention time [157,158]. To overcome these obstacles, therapeutic regimens are challenged to modify the molecular structure and formulation, such as the covalent incorporation of PEG and glycolic acid, or the construction of polymeric nanocomposites [159,160]. Kataoka et al. designed a (1,2-diaminocyclohexane)platinum(II) (DACHPt)-conjugating poly(ethylene glycol)-*b*-poly-(*L*-glutamic acid) (PEG-*b*-PGA) polymeric micelle with cyclic Arg-Gly-Asp (cRGD) ligand molecules for the targeted delivery of platinum therapeutic prodrugs to glioblastomas [86]. Compared to the polymeric micelle bearing nontargeted ligand (cyclic-Arg-Ala-Asp), cRGD-conjugated nanocarriers (cRGD/m) accumulated, accelerated, and possessed superior permeability from vessels into the tumor parenchyma (Figure 8A). The rapid accumulation of cRGD/m into tumor cells through an active internalization route should be responsible for the significantly improved tumor therapeutic effect in the treatment of U87MG glioblastoma.

Recently, Tang et al. reported a self-amplifying, therapeutic tumor-homing nanoplatform (A15-PGA-CA4) that works via a chain reaction mechanism [87]. The blood coagulation-targeting peptide (GNQEQVSPLTLLKXC, termed A15) was conjugated to poly(*L*-glutamic acid)-*graft*-maleimide poly(ethylene glycol)/combretastatin A4 (PGA-*g*-PEG-Mal/CA4) by thiol–maleimide “click chemistry” (Figure 8B). After intravenous injection, A15-PGA-CA4 released CA4 and initiated the chain reaction cycles: (1) intratumoral hemorrhage: CA4 selectively disrupted the established tumor blood vessels, leading to hemorrhage within treated tumor cells; (2) target blood coagulation factor XIIIa (FXIIIa) amplification: the hemorrhage triggered an intratumoral coagulation cascade effect, in which FXIII was activated into FXIIIa, causing targeted amplification; (3) blood clot binding: FXIIIa guided A15-PGA-CA4 in the blood stream to tumor tissues via binding to blood clot; (4) CA4 release in tumor sites: A15-PGA-CA4 continuously released CA4 in cancer cells and the next cycle started subsequently. A15- PGA-CA4 enhanced the content of the targeted FXIIIa via chain reaction. Owing to the superb targeting potency of A15- PGA-CA4, the tumor therapeutic effect against large C26 tumors was significantly improved. These impressive examples reaffirm the high specificity and efficacy of protein drugs which can be well exerted in the preparation of tumor-targeting biomaterials. Moreover, the exact chain reaction mechanism of A15- PGA-CA4 guided the combined cancer therapy based on co-delivery of chemical drugs and proteins.

### 3.8. Other PGA-Based Hybrids as Drug Delivery Nanovehicles

Owing to their intrinsic biodegradable and biocompatible characteristics, PGA has also been designed as a diverse kind of nanocomposite for biomedical applications [161,162,163,164,165]. For instance, Cheng et al. developed a tumor-targeting siRNA delivery nanoplatform based on a *α*-helical cell-penetrating polypeptide (PVBLG-8) and a random-coiled PGA [166]. The cationic and rigid PVBLG-8 exhibited weak siRNA condensation ability, whereas the anionic and flexible PGA acted as a stabilizer to entrap the siRNA within the molecular entanglement between PVBLG-8 and PGA. During circulation, the as-obtained nanonetwork displayed an anticipated serum stability and significantly increased tumor accumulation via the EPR effect. Vicent et al. designed a pH-responsive biodegradable polypeptide-corticosteroid conjugate (PGA-FLUO@HA-CP) for the topical treatment of psoriasis [167]. The PGA conjugation of fluocinolone acetonide (FLUO) contributed to penetration and drug exposure, thus improving targeting to the viable epidermis. Remarkably, the encapsulation of PGA-FLUO within the hyaluronic acid-poly(*L*-glutamate) cross polymer (HA-CP) vehicle further enhanced this targeting effect by boosting skin permeation. PGA-FLUO in synergy with HA-CP precisely delivered FLUO to the inflammatory skin layers, avoiding the potential side effects. Recently, Du et al. successfully prepared the bone-targeting polymeric vesicles for the effective treatment of postmenopausal osteoporosis [168]. These polymeric vesicles were self-assembled from poly(*ε*-caprolactone)_28_-*block*-poly[(*L*-glutamic acid)_7_-*stat*-(*L*-glutamic acid-alendronic acid)_4_] (PCL_28_-*b*-P[Glu_7_-*stat*-(Glu-ADA)_4_]) copolymers. The hydrophilic P[Glu_7_-*stat*-(Glu-ADA)_4_] chains acted as the coronas while the hydrophobic PCL chains served as the membranes which could encapsulate *β*-estradiol (E2) inside the vesicles. The conjugated ADA on the coronas enabled the vesicles with superior bone affinity and acted synergistically with E2 to increase bone mass and thus reached an enhanced osteoporosis treatment effect.

## 4. Conclusions and Perspectives

In this work, we have summarized recent vital advances of PGA-based nanomaterials in drug delivery. The inherent biodegradable, biocompatible, and ionic charging properties make PGA an attractive candidate for the construction of biomedical polymeric materials. The pensile carboxyl side groups of PGA provide abundant attachment sites for therapeutic drugs such as DOX, CPT, and Pt drugs, through either chemical incorporation or physical entrapment. PGA-based conjugates with defined structures and specific functions also have been prepared to deliver gas molecules or protein drugs. Owing to the synergistic therapeutic effects, diverse therapeutic techniques combined with chemotherapy have been widely reported by researchers. Moreover, the ionic-charged PGA have also been utilized in a variety of biomedical applications, such as antimicrobial complexes, vaccine adjuvants, tissue regeneration, and medical devices [169,170,171,172]. For example, clinical complications induced by tissue adhesion during surgery usually increase the degree of pain for patients [173,174]. Recently, Ko et al. designed a PGA-based anti-adhesion membrane conjugated with the anti-inflammatory drug ibuprofen for preventing tissue adhesion [175]. Owing to the proper hydrophilicity of PGA, this membrane exhibited superior wound coverage without the contraction. This study will certainly guide the future design and clinical applications of PGA-based nanomaterials.

Great efforts are required to achieve the translation of PGA-based nanomaterials from laboratory to clinic. PGA-based nanomaterials are confronted with two consistent challenges—enhanced therapeutic efficacy and scalable synthesized protocol. In addition to high stability during the systemic circulation, PGA-based nanomaterials should release the payload in the tumor sites. In consideration of the pendent carboxyl groups which endow PGA with pH-responsive property, various mono- or multi-stimuli responsive PGA-based nanomaterials have been proposed. For example, acid-labile hydrazone bonds and reduction-sensitive 2-azidoethyl disulfide linkage were utilized to construct dual-stimuli PGA-based prodrug with intracellular pH and redox responsiveness [91]. Furthermore, photo- and enzyme-responsive PGA conjugates also have been extensively reported, whereas further efforts are pressingly required to understand the mechanism of intratumor/intracellular release of payload [148,176]. High drug loading capacity and on-demand drug releasing ability at anticipated sites also play a crucial role in enhancing therapeutic efficiency. With the thrilling advances in NCA polymerization, synthetic polypeptides with well-defined structures and functionalities afford the novel building blocks for polymeric biomaterials. However, the property and function of these synthetic polypeptides are still far from matching the natural proteins. Therefore, it is a good opportunity to synthesize more functionalized polypeptides with predictable molecular weights, tunable monomer sequences, and controllable termini. On the other hand, the ingenious design of PGA-based nanomaterials usually involves multiple syntheses and purification procedures, which represent a formidable challenge for scalable and reproducible preparation of materials.

## Figures and Tables

**Table 1:** Summary of PGA-based nanocarriers with unique characteristics.

Main Polymers	Loading Drugs	Preparation Methods	Targeting Agents/Inhibitors	Ref.
mPEG-*b*-PGA	DOX	Electrostatic interaction & intermolecular hydrophobic stack	/	[71]
mPEG-*b*-PPLG	DOX	Chemical conjugation	/	[72]
PGA-(G-AGM)	DOX	Chemical conjugation	Aminoglutethimide	[73]
PEG-pH_e_-PGA	Cisplatin	Chelation interaction	/	[74]
PGA-*g*-Ve/PEG	Cisplatin DTX	Hydrophobic & chelation interaction	avb3 integrin targeting peptide	[75]
Mal-PEG_114_-*b*-PGA_12_	Cisplatin	Chelation interaction	Folic acid	[76]
PGA-*g*-mPEG	DOX CPT	Self-assembly	/	[77]
PEtO_x_-*b*-PGA	SN38	Chemical conjugation	/	[78]
PGA	CA4	Chemical conjugation	/	[79]
PGA	CA4	Chemical conjugation	Phosphoinositide 3-kinase gamma isoform	[80]
(PLL/PGA)_n_	NO	Layer by layer self-assembly	/	[81]
mPEG-*b*-PLG	DOX SO_2_	Self-assembly	/	[82]
PGA-*g*-mPEG	CA4 porphyrin	Chemical conjugation	/	[83]
mPEG-*g*-PGA	Cisplatin	Chelation interaction	Folic acid	[84]
PGA-*b*-PCL	DOX Gd(III)	Self-assembly	Folic acid	[85]
PEG-*b*-PGA	Cisplatin	Chelation interaction	Cyclic Arg-Gly-Asp	[86]
PGA-*g*-PEG-Mal	CA4	Chemical conjugation	Targeting peptide (GNQEQVSPLTLLKXC)	[87]

## Data Availability

Data supporting this publication are available from the corresponding authors.

## References

[1] Cabral H., Miyata K., Osada K., Kataoka K. (2018). Block copolymer micelles in nanomedicine applications. Chem. Rev..

[2] van der Meel R., Sulheim E., Shi Y., Kiessling F., Mulder W.J.M., Lammers T. (2019). Smart cancer nanomedicine. Nat. Nanotechnol..

[3] Khatoon N., Zhang Z., Zhou C., Chu M. (2022). Macrophage membrane coated nanoparticles: A biomimetic approach for enhanced and targeted delivery. Biomater. Sci..

[4] Yang J., Zhang X., Liu C., Wang Z., Deng L., Feng C., Tao W., Xu X., Cui W. (2021). Biologically modified nanoparticles as theranostic bionanomaterials. Prog. Polym. Sci..

[5] Tang Z., He C., Tian H., Ding J., Hsiao B.S., Chu B., Chen X. (2016). Polymeric nanostructured materials for biomedical applications. Prog. Polym. Sci..

[6] Zhang Y., Uthaman S., Song W., Eom K.H., Jeon S.H., Huh K.M., Babu A., Park I., Kim I. (2020). Multistimuli-responsive polymeric vesicles for accelerated drug release in chemo-photothermal therapy. ACS Biomater. Sci. Eng..

[7] Song W., Zhang Y., Yu D.-G., Tran C.H., Wang M., Varyambath A., Kim J., Kim I. (2021). Efficient synthesis of folate-conjugated hollow polymeric capsules for accurate drug delivery to cancer cells. Biomacromolecules.

[8] Zhao Z., Wang Z., Li G., Cai Z., Wu J., Wang L., Deng L., Cai M., Cui W. (2021). Injectable microfluidic hydrogel microspheres for cell and drug Delivery. Adv. Funct. Mater..

[9] Li H., Yin D., Li W., Tang Q., Zou L., Peng Q. (2021). Polydopamine-based nanomaterials and their potentials in advanced drug delivery and therapy. Colloids Surf. B Biointerfaces.

[10] Zhang M., Song W., Tang Y., Xu X., Huang Y., Yu D. (2022). Polymer-based nanofiber–nanoparticle hybrids and their medical applications. Polymers.

[11] Zhang Y., Li S., Xu Y., Zhang M., Huang Y., Liang Y., Chen Y., Ji W., Kim J.R., Song W. (2022). Engineering of hollow polymeric nanosphere-supported imidazolium-based ionic liquids with enhanced antimicrobial activities. Nano Res..

[12] Park S.-B., Sung M.-H., Uyama H., Han D.K. (2021). Poly(glutamic acid): Production, composites, and medical applications of the next-generation biopolymer. Prog. Polym. Sci..

[13] Shih I.-L., Van Y.-T., Shen M.H. (2004). Biomedical applications of chemically and microbiologically synthesized poly(glutamic acid) and poly(lysine). Mini Rev. Med. Chem..

[14] Alsaheb R.A.A., Othman N.Z., Malek R.A., Leng O.M., Aziz R., Enshasy H.A.E. (2016). Polyglutamic acid applications in pharmaceutical and biomedical industries. Der Pharm. Lett..

[15] Bajaj I., Singhal R. (2011). Poly(glutamic acid)–An emerging biopolymer of commercial interest. Bioresour. Technol..

[16] Shen Y., Fu X., Fu W., Li Z. (2015). Biodegradable stimuli-responsive polypeptide materials prepared by ring opening polymerization. Chem. Soc. Rev..

[17] Mazo A.R., Allison-Logan S., Karimi F., Chan N.J.-A., Qiu W., Duan W., O’ Brien-Simpson N.M., Qiao G.G. (2020). Ring opening polymerization of *α*-amino acids: Advances in synthesis, architecture and applications of polypeptides and their hybrids. Chem. Soc. Rev..

[18] Zhang Y., He P., Zhang P., Yi X., Xiao C., Chen X. (2021). Polypeptides–drug conjugates for anticancer therapy. Adv. Healthc. Mater..

[19] Manocha B., Margaritis A. (2010). Controlled release of doxorubicin from doxorubicin/*γ*-polyglutamic acid ionic complex. Nanomater..

[20] Li G., Wu J., Wang B., Yan S., Zhang K., Ding J., Yin J. (2015). Self-healing supramolecular self-assembled hydrogels based on poly (L-glutamic acid). Biomacromolecules.

[21] Laps S., Satish G., Brik A. (2021). Harnessing the power of transition metals in solid-phase peptide synthesis and key steps in the (semi) synthesis of proteins. Chem. Soc. Rev..

[22] Donovan A.J., Dowle J., Yang Y., Weiss M.A., Kent S.B.H. (2020). Total synthesis of bovine pancreatic trypsin inhibitor and the protein diastereomer [Gly37D-Ala]BPTI using Boc chemistry solid phase peptide synthesis. Peptide Sci..

[23] Luo Z., Guo Y., Liu J., Qiu H., Zhao M., Zou W., Li S. (2016). Microbial synthesis of poly-*γ*-glutamic acid: Current progress, challenges, and future perspectives. Biotechnol. Biofuels.

[24] Najar I., Das S. (2015). Poly-glutamic acid (PGA)-structure, synthesis, genomic organization and its application: A review. Int. J. Pharm. Sci. Res..

[25] Sirisansaneeyakul S., Cao M., Kongklom N., Chuensangjun C., Shi Z., Chisti Y. (2017). Microbial production of poly-*γ*-glutamic acid. J. Microbiol. Biotechnol..

[26] Zhou X., Li Z. (2018). Advances and biomedical applications of polypeptide hydrogels derived from α-amino acid *N*-carboxyanhydride (NCA) polymerizations. Adv. Healthc. Mater..

[27] Song Z., Tan Z., Cheng J. (2019). Recent advances and future perspectives of synthetic polypeptides from *N*-carboxyanhydrides. Macromolecules.

[28] Bu S., Yan S., Wang R., Xia P., Zhang K., Li G., Yin J. (2020). In situ precipitation of cluster and acicular hydroxyapatite onto porous poly (*γ*-benzyl-*l*-glutamate) microcarriers for bone tissue engineering. ACS Appl. Mater. Interfaces.

[29] Lavilla C., Byrne M., Heise A. (2016). Block-sequence-specific polypeptides from *α*-amino acid *N*-carboxyanhydrides: Synthesis and influence on polypeptide properties. Macromolecules.

[30] Kricheldorf H.R. (2006). Polypeptides and 100 years of chemistry of *α*-amino acid *N*-carboxyanhydrides. Angew. Chem. Int. Ed..

[31] Hadjichristidis N., Iatrou H., Pitsikalis M., Sakellariou G. (2009). Synthesis of well-defined polypeptide-based materials via the ring-opening polymerization of *α*-amino acid *N*-carboxyanhydrides. Chem. Rev..

[32] Deming T.J. (1997). Facile synthesis of block copolypeptides of defined architecture. Nature.

[33] Deming T.J. (1999). Cobalt and iron initiators for the controlled polymerization of *α*-amino acid-*N*-carboxyanhydrides. Macromolecules.

[34] Zhao W., Gnanou Y., Hadjichristidis N. (2015). From competition to cooperation: A highly efficient strategy towards well-defined (co)polypeptides. Chem. Commun..

[35] Zhao W., Gnanou Y., Hadjichristidis N. (2015). Fast and living ring-opening polymerization of *α*-amino acid *N*-carboxyanhydrides triggered by an “alliance” of primary and secondary amines at room temperature. Biomacromolecules.

[36] Lu H., Cheng J. (2007). Hexamethyldisilazane-mediated controlled polymerization of *α*-amino acid *N*-carboxyanhydrides. J. Am. Chem. Soc..

[37] Lu H., Cheng J. (2008). *N*-trimethylsilyl amines for controlled ring-opening polymerization of amino acid *N*-carboxyanhydrides and facile end group functionalization of polypeptides. J. Am. Chem. Soc..

[38] Baumgartner R., Fu H., Song Z., Lin Y., Cheng J. (2017). Cooperative polymerization of *α*-helices induced by macromolecular architecture. Nat. Chem..

[39] Chen C., Fu H., Baumgartner R., Song Z., Lin Y., Cheng J. (2019). Proximity-induced cooperative polymerization in “hinged” helical polypeptides. J. Am. Chem. Soc..

[40] Wu Y., Zhang D., Ma P., Zhou R., Hua L., Liu R. (2018). Lithium hexamethyldisilazide initiated superfast ring opening polymerization of *alpha*-amino acid *N*-carboxyanhydrides. Nat. Commun..

[41] Zhang Y., Liu R., Jin H., Song W., Augustin R., Kim I. (2018). Straightforward access to linear and cyclic polypeptides. Commun. Chem..

[42] Zhang Y., Song W., Li S., Kim D.-K., Kim J.H., Kim J.R., Kim I. (2020). Facile and scalable synthesis of topologically nanoengineered polypeptides with excellent antimicrobial activities. Chem. Commun..

[43] Coulembier O., Sanders D.P., Nelson A., Hollenbeck A.N., Horn H.W., Rice J.E., Fujiwara M., Dubois P., Hedrick J.L. (2009). Hydrogen-bonding catalysts based on fluorinated alcohol derivatives for living polymerization. Angew. Chem. Int. Ed..

[44] Kiesewetter M.K., Shin E.J., Hedrick J.L., Waymouth R.M. (2010). Organocatalysis: Opportunities and challenges for polymer synthesis. Macromolecules.

[45] Zhao W., Lv Y., Li J., Feng Z., Ni Y., Hadjichristidis N. (2019). Fast and selective organocatalytic ring-opening polymerization by fluorinated alcohol without a cocatalyst. Nat. Commun..

[46] Jacobs J., Pavlović D., Prydderch H., Moradi M.-A., Ibarboure E., Heuts J.P.A., Lecommandoux S., Heise A. (2019). Polypeptide nanoparticles obtained from emulsion polymerization of amino acid *N*-carboxyanhydrides. J. Am. Chem. Soc..

[47] Song Z., Fu H., Wang J., Hui J., Xue T., Pacheco L.A., Yan H., Baumgartner R., Wang Z., Xia Y. (2019). Synthesis of polypeptides via bioinspired polymerization of in situ purified *N*-carboxyanhydrides. Proc. Natl. Acad. Sci. USA.

[48] Zhang H., Nie Y., Zhi X., Du H., Yang J. (2017). Controlled ring-opening polymerization of *α*-amino acid *N*-carboxy-anhydride by frustrated amine/borane Lewis pairs. Chem. Commun..

[49] Jiang J., Zhang X., Fan Z., Du J. (2019). Ring-opening polymerization of *N*-carboxyanhydride-induced self-assembly for fabricating biodegradable polymer vesicles. ACS Macro Lett..

[50] Liu Y., Li D., Ding J., Chen X. (2020). Controlled synthesis of polypeptides. Chin. Chem. Lett..

[51] Zou J., Fan J., He X., Zhang S., Wang H., Wooley K.L. (2013). A facile glovebox-free strategy to significantly accelerate the syntheses of well-defined polypeptides by *N*-carboxyanhydride (NCA) ring-opening polymerizations. Macromolecules.

[52] Stukenkemper T., Paquez X., Verhoeven M., Hensen E.J., Dias A.A., Brougham D.F., Heise A. (2018). Polypeptide polymer brushes by light-induced surface polymerization of amino acid *N*-carboxyanhydrides. Macromol. Rapid Commun..

[53] Galluzzi L., Humeau J., Buqué A., Zitvogel L., Kroemer G. (2020). Immunostimulation with chemotherapy in the era of immune checkpoint inhibitors. Nat. Rev. Clin. Oncol..

[54] Bukowski K., Kciuk M., Kontek R. (2020). Mechanisms of multidrug resistance in cancer chemotherapy. Int. J. Mol. Sci..

[55] Wang X., Xuan Z., Zhu X., Sun H., Li J., Xie Z. (2020). Near-infrared photoresponsive drug delivery nanosystems for cancer photo-chemotherapy. J. Nanobiotechnol..

[56] Bai S., Jia D., Ma X., Liang M., Xue P., Kang Y., Xu Z. (2021). Cylindrical polymer brushes-anisotropic unimolecular micelle drug delivery system for enhancing the effectiveness of chemotherapy. Bioact. Mater..

[57] Sun M., Lee J., Chen Y., Hoshino K. (2022). Studies of nanoparticle delivery with in vitro bio-engineered microtissues. Bioact. Mater..

[58] Wang Y., Kohane D.S. (2017). External triggering and triggered targeting strategies for drug delivery. Nat. Rev. Mater..

[59] Badi N. (2017). Non-linear PEG-based thermoresponsive polymer systems. Prog. Polym. Sci..

[60] Yin Y., Chen G., Gong L., Ge K., Pan W., Li N., Machuki J.O., Yu Y., Geng D., Dong H. (2020). DNAzyme-powered three-dimensional DNA walker nanoprobe for detection amyloid *β*-peptide oligomer in living cells and in vivo. Anal. Chem..

[61] Balogun-Agbaje O.A., Odeniyi O.A., Odeniyi M.A. (2021). Drug delivery applications of poly-*γ*-glutamic acid. Future J. Pharm. Sci..

[62] Das M.P., Pandey G., Neppolian B., Das J. (2021). Design of poly-L-glutamic acid embedded mesoporous bioactive glass nanospheres for pH-stimulated chemotherapeutic drug delivery and antibacterial susceptibility. Colloids Surf. B Biointerfaces.

[63] Wang L., Chen S., Yu B. (2022). Poly-*γ*-glutamic acid: Recent achievements, diverse applications and future perspectives. Trends Food Sci. Technol..

[64] Wei M., Hsu Y.I., Asoh T.A., Sung M.H., Uyama H. (2021). Injectable poly (*γ*-glutamic acid)-based biodegradable hydrogels with tunable gelation rate and mechanical strength. J. Mater. Chem. B.

[65] Peng S.-F., Tseng M.T., Ho Y.-C., Wei M.-C., Liao Z.X., Sung H.-W. (2011). Mechanisms of cellular uptake and intracellular trafficking with chitosan/DNA/poly(*γ*-glutamic acid) complexes as a gene delivery vector. Biomaterials.

[66] Qi N., Tang B., Liu G., Liang X. (2017). Poly(*γ*-glutamic acid)-coated lipoplexes loaded with doxorubicin for enhancing the antitumor activity against liver tumors. Nanoscale Res. Lett..

[67] Tan J., Wang H., Xu F., Chen Y., Zhang M., Peng H., Sun X., Shen Y., Huang Y. (2017). Poly-*γ*-glutamic acid-based GGT-targeting and surface camouflage strategy for improving cervical cancer gene therapy. J. Mater. Chem. B.

[68] Zhang P., Li M., Xiao C., Chen X. (2021). Stimuli-responsive polypeptides for controlled drug delivery. Chem. Commun..

[69] Wang J., Xu W., Zhang N., Yang C., Xu H., Wang Z., Li B., Ding J., Chen X. (2021). X-ray-responsive polypeptide nanogel for concurrent chemoradiotherapy. J. Control. Release.

[70] Gao Z., He T., Zhang P., Li X., Zhang Y., Lin J., Hao J., Huang P., Cui J. (2020). Polypeptide-based theranostics with tumor-microenvironment-activatable cascade reaction for chemo-ferroptosis combination therapy. ACS Appl. Mater. Interfaces.

[71] Li M., Song W., Tang Z., Lv S., Lin L., Sun H., Li Q., Yang Y., Hong H., Chen X. (2013). Nanoscaled poly(*L*-glutamic acid)/doxorubicin-amphiphile complex as pH-responsive drug delivery system for effective treatment of nonsmall cell lung cancer. ACS Appl. Mater. Interfaces.

[72] Zhang Y., Ding J., Li M., Chen X., Xiao C., Zhuang X., Huang Y., Chen X. (2016). One-step “click chemistry”-synthesized cross-linked prodrug nanogel for highly selective intracellular drug delivery and upregulated antitumor efficacy. ACS Appl. Mater. Interfaces.

[73] Arroyo-Crespo J.J., Armiñán A., Charbonnier D., Balzano-Nogueira L., Huertas-López F., Martí C., Tarazona S., Forteza J., Conesa A., Vicent M.J. (2018). Tumor microenvironment-targeted poly-*L*-glutamic acid-based combination conjugate for enhanced triple negative breast cancer treatment. Biomaterials.

[74] Jiang Z., Feng X., Zou H., Xu W., Zhuang X. (2021). Poly(*l*-glutamic acid)-cisplatin nanoformulations with detachable PEGylation for prolonged circulation half-life and enhanced cell internalization. Bioact. Mater..

[75] Song W., Tang Z., Zhang D., Zhang Y., Yu H., Li M., Lv S., Sun H., Deng M., Chen X. (2014). Anti-tumor efficacy of c(RGDfK)-decorated polypeptide-based micelles co-loaded with docetaxel and cisplatin. Biomaterials.

[76] Shirbin S.J., Ladewig K., Fu Q., Klimak M., Zhang X., Duan W., Qiao G.G. (2015). Cisplatin-induced formation of biocompatible and biodegradable polypeptide-based vesicles for targeted anticancer drug delivery. Biomacromolecules.

[77] Li Y., Yang H., Yao J., Yu H., Chen X., Zhang P., Xiao C. (2018). Glutathione-triggered dual release of doxorubicin and camptothecin for highly efficient synergistic anticancer therapy. Colloids Surf. B Biointerfaces.

[78] Salmanpour M., Yousefi G., Samani S.M., Mohammdai S., Anbardar M.H., Tamaddon A. (2019). Nanoparticulate delivery of irinotecan active metabolite (SN38) in murine colorectal carcinoma through conjugation to poly (2-ethyl 2-oxazoline)-*b*-poly (*L*-glutamic acid) double hydrophilic copolymer. Eur. J. Pharm. Sci..

[79] Liu T., Zhang D., Song W., Tang Z., Zhu J., Ma Z., Wang X., Chen X., Tong T. (2017). A poly(*l*-glutamic acid)-combretastatin A4 conjugate for solid tumor therapy: Markedly improved therapeutic efficiency through its low tissue penetration in solid tumor. Acta Biomater..

[80] Qin H., Yu H., Sheng J., Zhang D., Shen N., Liu L., Tang Z., Chen X. (2019). PI3Kgamma inhibitor attenuates immunosuppressive effect of poly(*l*-Glutamic Acid)-combretastatin A4 conjugate in metastatic breast cancer. Adv. Sci..

[81] Park K., Jeong H., Tanum J., Yoo J.-C., Hong J. (2019). Poly-*L*-lysine/poly-*L*-glutamic acid-based layer-by-layer self-assembled multilayer film for nitric oxide gas delivery. J. Ind. Eng. Chem..

[82] Zhang Y., Shen W., Zhang P., Chen L., Xiao C. (2022). GSH-triggered release of sulfur dioxide gas to regulate redox balance for enhanced photodynamic therapy. Chem. Commun..

[83] Yu H., Bao Y., Xu C., Chen L., Tang Z. (2021). Poly(*L*-glutamic Acid)-drug conjugates for chemo- and photodynamic combination therapy. Macromol. Biosci..

[84] Feng B., Xu Z., Zhou F., Yu H., Sun Q., Wang D., Tang Z., Yu H., Yin Q., Zhang Z. (2015). Near infrared light-actuated gold nanorods with cisplatin–polypeptide wrapping for targeted therapy of triple negative breast cancer. Nanoscale.

[85] Liu Q., Chen S., Chen J., Du J. (2015). An asymmetrical polymer vesicle strategy for significantly improving T1 MRI sensitivity and cancer-targeted drug delivery. Macromolecules.

[86] Miura Y., Takenaka T., Toh K., Wu S., Nishihara H., Kano M.R., Nomoto Y.I.T., Matsumoto Y., Koyama H., Cabral H. (2013). Cyclic RGD-linked polymeric micelles for targeted delivery of platinum anticancer drugs to glioblastoma through the blood-brain tumor barrier. ACS Nano.

[87] Wang Y., Shen N., Wang Y., Zhang Y., Tang Z., Chen X. (2021). Self-amplifying nanotherapeutic drugs homing to tumors in a manner of chain reaction. Adv. Mater..

[88] Tacar O., Sriamornsak P., Dass C.R. (2013). Doxorubicin: An update on anticancer molecular action, toxicity and novel drug delivery systems. J. Pharm. Pharmacol..

[89] Zhang X., Xi Z., Machuki J.O., Luo J., Yang D., Li J., Cai W., Yang Y., Zhang L., Tian J. (2019). Gold cube-in-cube based oxygen nanogenerator: A theranostic nanoplatform for modulating tumor microenvironment for precise chemo-phototherapy and multimodal imaging. ACS Nano.

[90] Zhang X., Zhao M., Cao N., Qin W., Zhao M., Wu J., Lin D. (2020). Construction of a tumor microenvironment pH-responsive cleavable PEGylated hyaluronic acid nano-drug delivery system for colorectal cancer treatment. Biomater. Sci..

[91] Akao T., Kimura T., Hirofuji Y.-S., Matsunaga K., Imayoshi R., Nagao J.-I., Cho T., Matsumoto H., Ohtono S., Ohno J. (2010). A poly(*γ*-glutamic acid)–amphiphile complex as a novel nanovehicle for drug delivery system. J. Drug Target..

[92] Zhang Y., Xiao C., Ding J., Li M., Chen X., Tang Z., Zhuang X., Chen X. (2016). A comparative study of linear, Y-shaped and linear-dendritic methoxy poly(ethylene glycol)-*block*-polyamidoamine-*block*-poly(*l*-glutamic acid) block copolymers for doxorubicin delivery in vitro and in vivo. Acta Biomater..

[93] Qu J., Wang R., Peng S., Shi M., Yang S., Luo J., Lin J., Zhou Q. (2019). Stepwise dual pH and redox-responsive cross-linked polypeptide nanoparticles for enhanced cellular uptake and effective cancer therapy. J. Mater. Chem. B.

[94] Yang S., Zhu F., Wang Q., Liang F., Qu X., Gan Z., Yang Z. (2016). Nano-rods of doxorubicin with poly(*L*-glutamic acid) as a carrier-free formulation for intratumoral cancer treatment. J. Mater. Chem. B.

[95] Duro-Castano A., Sousa-Herves A., Armiñán A., Charbonnier D., Arroyo-Crespo J.J., Wedepohl S., Calderón M., Vicent M.J. (2021). Polyglutamic acid-based crosslinked doxorubicin nanogels as an anti-metastatic treatment for triple negative breast cancer. J. Control. Release.

[96] Ding J., Xu W., Zhang Y., Sun C., Liu D., Zhu X., Chen X. (2013). Self-reinforced endocytoses of smart polypeptide nanogels for “on-demand” drug delivery. J. Control. Release.

[97] Jaimes-Aguirre L., Morales-Avila E., Ocampo-García B.E., Medina L.A., López-Téllez G., Gibbens-Bandala B.V., Izquierdo-Sánchez V. (2017). Biodegradable poly (*D*, *L*-lactide-*co*-glycolide)/poly (*L*-*γ*-glutamic acid) nanoparticles conjugated to folic acid for targeted delivery of doxorubicin. Mater. Sci. Eng. C.

[98] Wang C., Wang B., Zou S., Wang B., Liu G., Zhang F., Wang Q., He Q., Zhang L. (2021). Cyclo-*γ*-polyglutamic acid-coated dual-responsive nanomicelles loaded with doxorubicin for synergistic chemo-photodynamic therapy. Biomater. Sci..

[99] Tyrrell Z.L., Shen Y., Radosz M. (2010). Fabrication of micellar nanoparticles for drug delivery through the self-assembly of block copolymers. Prog. Polym. Sci..

[100] Lv S., Li M., Tang Z., Song W., Sun H., Liu H., Chen X. (2013). Doxorubicin-loaded amphiphilic polypeptide-based nanoparticles as an efficient drug delivery system for cancer therapy. Acta Biomater..

[101] Chen P., Qiu M., Deng C., Meng F., Zhang J., Cheng R., Zhong Z. (2015). pH-Responsive chimaeric pepsomes based on asymmetric poly(ethylene glycol)-*b*-poly(*l*-leucine)-*b*-poly(*l*-glutamic acid) triblock copolymer for efficient loading and active intracellular delivery of doxorubicin hydrochloride. Biomacromolecules.

[102] Bae Y., Fukushima S., Harada A., Kataoka K. (2003). Design of environment-sensitive supramolecular assemblies for intracellular drug delivery: Polymeric micelles that are responsive to intracellular pH change. Angew. Chem. Int. Ed..

[103] Li J., Du N., Tan Y., Hsu H.Y., Tan C., Jiang Y. (2021). Conjugated polymer nanoparticles based on copper coordination for real-time monitoring of pH-responsive drug delivery. ACS Appl. Bio Mater..

[104] Shen H., Liu Q., Liu D., Yu S., Wang X., Yang M. (2021). Fabrication of doxorubicin conjugated methoxy poly (ethylene glycol)-*block*-poly (*ε*-caprolactone) nanoparticles and study on their in vitro antitumor activities. J. Biomater. Sci. Polym. Ed..

[105] Sang X., Yang Q., Wen Q., Zhang L., Ni C. (2019). Preparation and controlled drug release ability of the poly [*N*-isopropylacryamide-*co*-allyl poly (ethylene glycol)]-*b*-poly (*γ*-benzyl-l-glutamate) polymeric micelles. Mater. Sci. Eng. C.

[106] Pisarevsky E., Blau R., Epshtein Y., Ben-Shushan D., Eldar-Boock A., Tiram G., Koshrovski-Michael S., Scomparin A., Pozzi S., Krivitsky A. (2020). Rational design of polyglutamic acid delivering an optimized combination of drugs targeting mutated BRAF and MEK in melanoma. Adv. Ther..

[107] Arroyo-Crespo J.J., Deladriere C., Nebot V.J., Charbonnier D., Masiá E., Paul A., James C., Armiñán A., Vicent M.J. (2018). Anticancer activity driven by drug linker modification in a polyglutamic acid-based combination-drug conjugate. Adv. Funct. Mater..

[108] Wang R., He D., Wang H., Wang J., Yu Y., Chen Q., Sun C., Shen Y., Tu J., Xiong Y. (2020). Redox-sensitive polyglutamic acid-platinum (IV) prodrug grafted nanoconjugates for efficient delivery of cisplatin into breast tumor. Nanomed. Nanotechnol. Biol. Med..

[109] Xu Y., Han X., Li Y., Min H., Zhao X., Zhang Y., Qi Y., Shi J., Qi S., Bao Y. (2019). Sulforaphane mediates glutathione depletion via polymeric nanoparticles to restore cisplatin chemosensitivity. ACS Nano.

[110] Xiang J., Li Y., Zhang Y., Wang G., Xu H., Zhou Z., Tang J., Shen Y. (2021). Polyphenol-cisplatin complexation forming core-shell nanoparticles with improved tumor accumulation and dual-responsive drug release for enhanced cancer chemotherapy. J. Control. Release.

[111] Reedijk J. (2003). New clues for platinum antitumor chemistry: Kinetically controlled metal binding to DNA. Proc. Natl. Acad. Sci. USA.

[112] Koberle B., Tomicic M.T., Usanova S., Kaina B. (2010). Cisplatin resistance: Preclinical findings and clinical implications. Biochim. Biophys. Acta Rev. Cancer.

[113] Gao Z., Zhang Z., Guo J., Hao J., Zhang P., Cui J. (2020). Polypeptide nanoparticles with pH-sheddable PEGylation for improved drug delivery. Langmuir.

[114] Ahmad Z., Tang Z., Shah A., Lv S., Zhang D., Zhang Y., Chen X. (2014). Cisplatin loaded methoxy poly (ethylene glycol)-*block*-poly (*L*-glutamic acid-co-*L*-Phenylalanine) nanoparticles against human breast cancer cell. Macromol. Biosci..

[115] Ding N., Zhao Z., Yin N., Xu Y., Yin T., Gou J., He H., Wang Y., Zhang Y., Tang X. (2021). Co-delivery of gemcitabine and cisplatin via poly(*L*-glutamic acid)-*g*-methoxy poly (ethylene glycol) micelle to improve the in vivo stability and antitumor effect. Pharm. Res..

[116] Nishiyama N., Yokoyama M., Aoyagi T., Okano T., Sakurai Y., Kataoka K. (1999). Preparation and characterization of self-assembled polymer−metal complex micelle from cis-dichlorodiammineplatinum(II) and poly(ethylene glycol)−poly(*α*,*β*-aspartic acid) block copolymer in an aqueous medium. Langmuir.

[117] Endo K., Ueno T., Kondo S., Wakisaka N., Murono S., Ito M., Kataoka K., Kato Y., Yoshizaki T. (2013). Tumor-targeted chemotherapy with the nanopolymer-based drug NC-6004 for oral squamous cell carcinoma. Cancer Sci..

[118] Ueno T., Endo K., Hori K., Ozaki N., Tsuji A., Kondo S., Wakisaka N., Murono S., Kataoka K., Kato Y. (2014). Assessment of antitumor activity and acute peripheral neuropathy of 1,2-diaminocyclohexane platinum (II)-incorporating micelles (NC-4016). Int. J. Nanomed..

[119] Sun Q., Zhou Z., Qiu N., Shen Y. (2017). Rational design of cancer nanomedicine: Nanoproperty integration and synchronization. Adv. Mater..

[120] Mi P., Cabral H., Kataoka K. (2020). Ligand-installed nanocarriers toward precision therapy. Adv. Mater..

[121] Song W., Tang Z., Shen N., Yu H., Jia Y., Zhang D., Jiang J., He C., Tian H., Chen X. (2016). Combining disulfiram and poly(*L*-glutamic acid)-cisplatin conjugates for combating cisplatin resistance. J. Control. Release.

[122] Wall M.E., Wani M.C., Cook C.E., Palmer K.H., McPhail A.T., Sim G.A. (1966). Plant antitumor agents. I. the isolation and structure of camptothecin, a novel alkaloidal leukemia and tumor inhibitor from camptotheca acuminata. J. Am. Chem. Soc..

[123] Yao X., Mu J., Zeng L., Lin J., Nie Z., Jiang X., Huang P. (2019). Stimuli-responsive cyclodextrin-based nanoplatforms for cancer treatment and theranostics. Mater. Horiz..

[124] Zhang D., Li L., Ji X., Gao Y. (2019). Intracellular GSH-responsive camptothecin delivery systems. New J. Chem..

[125] Patil A.R., Nimbalkar M.S., Patil P.S., Chougale A.D., Patil P.B. (2020). Controlled release of poorly water soluble anticancerous drug camptothecin from magnetic nanoparticles. Mater. Today Proc..

[126] Kim S.H., Kaplan J.A., Sun Y., Shieh A., Sun H.-L., Croce P.C.M., Grinstaff P.M.W. (2015). The self-assembly of anticancer camptothecin–dipeptide nanotubes: A minimalistic and high drug loading approach to increased efficacy. Chem. Eur. J..

[127] Bhatt R., de Vries P., Tulinsky J., Bellamy G., Baker B., Singer J.W., Klein P. (2003). Synthesis and in vivo antitumor activity of poly(*l*-glutamic acid) conjugates of 20(S)-camptothecin. J. Med. Chem..

[128] Singer J.W., Bhatt R., Tulinsky J., Buhler K.R., Heasley E., Klein P., de Vries P. (2001). Water-soluble poly-(*l*-glutamic acid)–Gly-camptothecin conjugates enhance camptothecin stability and efficacy in vivo. J. Control. Release.

[129] Li Y., Lu H., Liang S., Xu S. (2019). Dual stable nanomedicines prepared by cisplatin-crosslinked camptothecin prodrug micelles for effective drug delivery. ACS Appl. Mater. Interfaces.

[130] Wu D., Li Y., Zhu L., Zhang W., Xu S., Yang Y., Yan Y., Yan Q., Yang G. (2021). A biocompatible superparamagnetic chitosan-based nanoplatform enabling targeted SN-38 delivery for colorectal cancer therapy. Carbohydr. Polym..

[131] Siemann D.W., Chaplin D.J., Horsman M.R. (2004). Vascular-targeting therapies for treatment of malignant disease. Cancer.

[132] Song W., Tang Z., Zhang D., Li M., Gu J., Chen X. (2016). A cooperative polymeric platform for tumor-targeted drug delivery. Chem. Sci..

[133] Galbraith S.M., Chaplin D.J., Lee F., Stratford M.R.L., Locke R.J., Vojnovic B., Tozer G.M. (2001). Effects of combretastatin A4 phosphate on endothelial cell morphology in vitro and relationship to tumour vascular targeting activity in vivo. Anticancer Res..

[134] Ma X., Dong X., Zan C., Sun Y., Qian J., Yuan Y., Liu C. (2019). Polyglutamic acid-coordinated assembly of hydroxyapatite nanoparticles for synergistic tumor-specific therapy. Nanoscale.

[135] Wang Y., Yu H., Zhang D., Wang G., Song W., Liu Y., Ma S., Tang Z., Liu Z., Sakurai K. (2019). Co-administration of combretastatin A4 nanoparticles and sorafenib for systemic therapy of hepatocellular carcinoma. Acta Biomater..

[136] Yang S., Tang Z., Hu C., Zhang D., Shen N., Yu H., Chen X. (2019). Selectively potentiating hypoxia levels by combretastatin A4 nanomedicine: Toward highly enhanced hypoxia-activated prodrug tirapazamine therapy for metastatic tumors. Adv. Mater..

[137] Yu H., Shen N., Bao Y., Chen L., Tang Z. (2020). Tumor regression and potentiation of polymeric vascular disrupting therapy through reprogramming of a hypoxia microenvironment with temsirolimus. Biomater. Sci..

[138] Zhang H., Xie M., Chen H., Bavi S., Sohail M., Bavi R. (2021). Gas-mediated cancer therapy. Environ. Chem. Lett..

[139] Chen L., Zhou S.-F., Su L., Song J. (2019). Gas-mediated cancer bioimaging and therapy. ACS Nano.

[140] Yu L., Hu P., Chen Y. (2018). Gas-generating nanoplatforms: Material chemistry, multifunctionality, and gas therapy. Adv. Mater..

[141] Wang Y., Yang T., He Q. (2020). Strategies for engineering advanced nanomedicines for gas therapy of cancer. Natl. Sci. Rev..

[142] Wan S.-S., Zeng J.-Y., Cheng H., Zhang X.-Z. (2018). ROS-induced NO generation for gas therapy and sensitizing photodynamic therapy of tumor. Biomaterials.

[143] Ding Y., Ma Y., Du C., Wang C., Chen T., Wang Y., Wang J., Yao Y., Dong C.-M. (2021). NO-releasing polypeptide nanocomposites reverse cancer multidrug resistance via triple therapies. Acta Biomater..

[144] Shen W., Liu W., Yang H., Zhang P., Xiao C., Chen X. (2018). A glutathione-responsive sulfur dioxide polymer prodrug as a nanocarrier for combating drug-resistance in cancer chemotherapy. Biomaterials.

[145] Li W., Han P., Chen Y., Guo Y., Li D., Wu Y., Yue Y., Chu M. (2018). Drug-loaded polymer-coated graphitic carbon nanocages for highly efficient in vivo near-infrared laser-induced synergistic therapy through enhancing initial temperature. ACS Appl. Mater..

[146] Zhang X., Machuki J.O., Pan W., Cai W., Xi Z., Shen F., Zhang L., Yang Y., Gao F., Guan M. (2020). Carbon nitride hollow theranostic nanoregulators executing laser-activatable water splitting for enhanced ultrasound/fluorescence imaging and cooperative phototherapy. ACS Nano.

[147] Pan W., Dai C., Li Y., Yin Y., Gong L., Machuki J.O., Yang Y., Qiu S., Guo K., Gao F. (2020). PRP-chitosan thermoresponsive hydrogel combined with black phosphorus nanosheets as injectable biomaterial for biotherapy and phototherapy treatment of rheumatoid arthritis. Biomaterials.

[148] Xiong R., Hua D., Van Hoeck J., Berdecka D., Léger L., De Munter S., Fraire J.C., Raes L., Harizaj A., Sauvage F. (2021). Photothermal nanofibres enable safe engineering of therapeutic cells. Nat. Nanotechnol..

[149] Yorseng K., Siengchin S., Ashok B., Rajulu A.V. (2020). Nanocomposite egg shell powder with in situ generated silver nanoparticles using inherent collagen as reducing agent. J. Bioresour. Bioprod..

[150] Liu P., Yang W., Shi L., Zhang H., Xu Y., Wang P., Zhang G., Chen W.R., Zhang B., Wang X. (2019). Concurrent photothermal therapy and photodynamic therapy for cutaneous squamous cell carcinoma by gold nanoclusters under a single NIR laser irradiation. J. Mater. Chem. B.

[151] Bao Y., Yu H., Zhang Y., Chen L. (2021). Comparative study of two poly(amino acid)-based photosensitizer-delivery systems for photodynamic therapy. Int. J. Biol. Macromol..

[152] Xu C., Wang Y., Yu H., Tian H., Chen X. (2018). Multifunctional theranostic nanoparticles derived from fruit-extracted anthocyanins with dynamic disassembly and elimination abilities. ACS Nano.

[153] Varanko A., Saha S., Chikoti A. (2020). Recent trends in protein and peptide-based biomaterials for advanced drug delivery. Adv. Drug Deliv. Rev..

[154] Bajrachaya R., Song J.G., Back S.Y., Han H.-K. (2019). Recent advancements in non-invasive formulations for protein drug delivery. Comput. Struct. Biotechnol..

[155] Zhao L., Skwarczynski M., Toth I. (2019). Polyelectrolyte-based platforms for the delivery of peptides and proteins. ACS Biomater. Sci. Eng..

[156] Rostami E. (2020). Progresses in targeted drug delivery systems using chitosan nanoparticles in cancer therapy: A mini-review. J. Drug Deliv. Sci. Technol..

[157] Walsh G. (2014). Biopharmaceutical benchmarks 2014. Nat. Biotechnol..

[158] Hou Y., Lu H. (2019). Protein PEPylation: A new paradigm of protein–polymer conjugation. Bioconjugate Chem..

[159] Li X., Xia W., Quan G., Huang Y., Bai X., Yu F., Xu Q., Qin W., Liu D., Pan X. (2020). Poly(*L*-glutamic acid)-based brush copolymers: Fabrication, self-assembly, and evaluation as efficient nanocarriers for cationic protein drug delivery. AAPS PharmSciTech.

[160] Wu X., He C., Wu Y., Chen X., Cheng J. (2015). Nanogel-incorporated physical and chemical hybrid gels for highly effective chemo–protein combination therapy. Adv. Funct. Mater..

[161] Nguyen H.T., Phung C.D., Tran T.H., Pham T.T., Pham L.M., Nguyen T.T., Jeong J.-H., Choi H.-G., Ku S.K., Yong C.S. (2021). Manipulating immune system using nanoparticles for an effective cancer treatment: Combination of targeted therapy and checkpoint blockage miRNA. J. Control. Release.

[162] Si X., Ma S., Xu Y., Zhang D., Shen N., Yu H., Zhang Y., Song W., Tang Z., Chen X. (2020). Hypoxia-sensitive supramolecular nanogels for the cytosolic delivery of ribonuclease a as a breast cancer therapeutic. J. Control. Release.

[163] Ryu K., Lee M.K., Park J., Kim T. (2018). pH-Responsive charge-conversional poly(ethylene imine)–poly(*l*-lysine)–poly(*l*-glutamic acid) with self-assembly and endosome buffering ability for gene delivery systems. ACS Appl. Bio. Mater..

[164] Hadi M.M., Nesbitt H., Masood H., Sciscione F., Patel S., Ramesh B.S., Emberton M., Callan J.F., MacRobert A., McHale A.P. (2021). Investigating the performance of a novel pH and cathepsin B sensitive, stimulus-responsive nanoparticle for optimised sonodynamic therapy in prostate cancer. J. Control. Release.

[165] Ashok B., Hariram N., Siengchin S., Rajulu A.V. (2020). Modification of tamarind fruit shell powder with in situ generated copper nanoparticles by single step hydrothermal method. J. Bioresour. Bioprod..

[166] Liu Y., Song Z., Zheng N., Nagasaka K., Yin L., Cheng J. (2018). Systemic siRNA delivery to tumors by cell-penetrating *α*-helical polypeptide-based metastable nanoparticles. Nanoscale.

[167] Dolz-Pérez I., Sallam M.A., Masiá E., Morelló-Bolumar D., del Caz M.D., Graff P., Abdelmonsif D., Hedtrich S., Nebot V.J., Vicent M.J. (2020). Polypeptide-corticosteroid conjugates as a topical treatment approach to psoriasis. J. Control. Release.

[168] Zhou X., Cornel E.J., Fan Z., He S., Du J. (2021). Bone-targeting polymer vesicles for effective therapy of osteoporosis. Nano Lett..

[169] Tong Z., Yang J., Lin L., Wang R., Cheng B., Chen Y., Tang L., Chen J., Ma X. (2019). In situ synthesis of poly (*γ*-glutamic acid)/alginate/AgNP composite microspheres with antibacterial and hemostatic properties. Carbohydr. Polym..

[170] Pathinayake P.S., Gayan Chathuranga W.A., Lee H.C., Chowdhury M.Y., Sung M.H., Lee J.S., Kim C.J. (2018). Inactivated enterovirus 71 with poly-*γ*-glutamic acid/Chitosan nano particles (PC NPs) induces high cellular and humoral immune responses in BALB/c mice. Arch. Virol..

[171] Xu T., Yang R., Ma X., Chen W., Liu S., Liu X., Cai X., Xu H., Chi B. (2019). Bionic Poly (*γ*-Glutamic Acid) electrospun fibrous scaffolds for preventing hypertrophic scars. Adv. Healthc. Mater..

[172] Zhang L., Ma Y., Pan X., Chen S., Zhuang H., Wang S. (2018). A composite hydrogel of chitosan/heparin/poly (*γ*-glutamic acid) loaded with superoxide dismutase for wound healing. Carbohy. Polym..

[173] Diamond M.P., Freeman M.L. (2001). Clinical implications of postsurgical adhesions. Hum. Reprod. Update.

[174] Kim J.Y., Cho W.J., Kim J.H., Lim S.H., Kim H.J., Lee Y.W., Kwon S.W. (2013). Efficacy and safety of hyaluronate membrane in the rabbit cecum-abdominal wall adhesion model. J. Korean Surg. Soc..

[175] Ko Y.G., Yoon K.H., Park C., Sung M.H., Kwon O.K., Ahn C.H., Kim Y.J., Kwon O.H. (2007). Preparation and evaluation of poly (γ-glutamic acid)-based anti-adhesion membranes. Key Eng. Mater..

[176] Jiang J., Shen N., Ci T., Tang Z., Gu Z., Li G., Chen X. (2019). Combretastatin A4 nanodrug-Induced MMP9 amplification boosts tumor-selective release of doxorubicin prodrug. Adv. Mater..

